# Impairment of type H vessels by NOX2-mediated endothelial oxidative stress: critical mechanisms and therapeutic targets for bone fragility in streptozotocin-induced type 1 diabetic mice

**DOI:** 10.7150/thno.50907

**Published:** 2021-01-30

**Authors:** Xiao-Fan Hu, Geng Xiang, Tian-Ji Wang, Yu-Bo Ma, Yang Zhang, Ya-Bo Yan, Xiong Zhao, Zi-Xiang Wu, Ya-Fei Feng, Wei Lei

**Affiliations:** 1 Department of Orthopedics, Xijing Hospital, The Fourth Military Medical University, Xi'an 710032, China.; 2 Department of Dermatology and Venereology, Peking University First Hospital, Research Center for Medical Mycology, Peking University, Beijing 100034, China.

**Keywords:** Diabetic bone fragility, Type H vessels, Endothelial damage, Oxidative stress, NADPH oxidase 2.

## Abstract

**Rationale**: Mechanisms underlying the compromised bone formation in type 1 diabetes mellitus (T1DM), which causes bone fragility and frequent fractures, remain poorly understood. Recent advances in organ-specific vascular endothelial cells (ECs) identify type H blood vessel injury in the bone, which actively direct osteogenesis, as a possible player.

**Methods**: T1DM was induced in mice by streptozotocin (STZ) injection in two severity degrees. Bony endothelium, the coupling of angiogenesis and osteogenesis, and bone mass quality were evaluated. Insulin, antioxidants, and NADPH oxidase (NOX) inhibitors were administered to diabetic animals to investigate possible mechanisms and design therapeutic strategies.

**Results**: T1DM in mice led to the holistic abnormality of the vascular system in the bone, especially type H vessels, resulting in the uncoupling of angiogenesis and osteogenesis and inhibition of bone formation. The severity of osteopathy was positively related to glycemic levels. These pathological changes were attenuated by early-started, but not late-started, insulin therapy. ECs in diabetic bones showed significantly higher levels of reactive oxygen species (ROS) and NOX 1 and 2. Impairments of bone vessels and bone mass were effectively ameliorated by treatment with anti-oxidants or NOX2 inhibitors, but not by a NOX1/4 inhibitor. GSK2795039 (GSK), a NOX2 inhibitor, significantly supplemented the insulin effect on the diabetic bone.

**Conclusions**: Diabetic osteopathy could be a chronic microvascular complication of T1DM. The impairment of type H vessels by NOX2-mediated endothelial oxidative stress might be an important contributor that can serve as a therapeutic target for T1DM-induced osteopathy.

## Introduction

Fragility fractures are a frequent but under-appreciated complication of type 1 diabetes mellitus (T1DM) 1. There is a 6-7 times higher risk of fractures 2, and delayed or failed fracture healing in the T1DM population, which results in poor outcomes and high mortality 1. These diabetic bone diseases derive mainly from compromised bone formation and subsequent bone loss 1, 3, but the underlying pathogenesis mechanisms remain elusive. Current osteoporosis therapies aimed at inhibiting bone resorption are inadequate. Despite improvements in insulin replacement therapy, osteopenia and high fracture rates still exist in the majority of T1DM patients 1, 4. A better understanding of the mechanisms underlying bone formation inhibition in T1DM is needed to provide new vistas for therapeutic potential.

Diabetes causes vascular diseases affecting nearly all vascular types and sizes 5, 6. Vascular impairment has been demonstrated to be a critical player in many diabetic pathological changes, such as retinopathy and nephropathy, considered as vascular complications of diabetes 5, 7. Bone is an organ rich in the vascular network; however, the influence of diabetes on bone blood vessels and its role in diabetic osteopathy remain poorly understood 7. Vascular complications have emerged as predictors of fractures in most studies of T1DM 1, 4. Increasing evidence indicates a direct deleterious effect of microangiopathy on the bone 7, but is still open to question and it remains uncertain whether diabetic osteopathy can be classified as a chronic, microvascular complication of diabetes 7, 8. Therefore, confirmation of whether microangiopathy is central to or a contributory factor to diabetic osteopathy is crucial for the screening and treatment of diabetic bone diseases 7.

Endothelial cells (ECs) that line capillaries have long been perceived only as passive conduits for blood. This stereotype, however, has been challenged by increasing evidence indicating the critical role of organ-specific ECs as important regulators that direct the development, metabolism, and homeostasis of organs 9, 10. Kusumbe *et al.* 11 have demonstrated that vascular growth in bone involves a specialized, tissue-specific form of angiogenesis coupled with osteogenesis and that bone vasculature contains specialized type H and type L EC subtypes. Type H ECs, characterized by high expression of CD31 and endomucin (Emcn), serve as a key regulator of bone formation by secreting angiocrine factors and mediating the coupling of angiogenesis and osteogenesis. It has been proposed that human type H vessels might be a sensitive biomarker of bone mass 12. The reduction of type H capillaries has been implicated in the bone loss in mouse models of aging and postmenopausal osteoporosis 11, 13-15, but the changes of type H vessels in diabetes and its relationship with diabetic osteopathy are still unknown. The impairment of type H vessels could be a critical reason for poor bone formation and increased bone fragility in T1DM, and its underlying pathophysiological mechanisms remain to be elucidated.

In the present study, we found that early-onset T1DM led to an overall abnormality of bone blood vessels, especially type H vessels, resulting in the uncoupling of angiogenesis and osteogenesis and compromised bone growth. The extent of these pathological changes was related to glycemic levels, and early-started sufficient insulin therapy for glycemic control was needed to avoid the irreversible vascular lesions in bone. Endothelial oxidative stress might be an important contributor to the diabetic impairment of bone vessels, with NADPH oxidase 2 (NOX2) serving as a crucial molecular therapeutic target and NOX2 inhibitors as potential efficacious drugs.

## Methods

### Animal model

**Figure 1:**
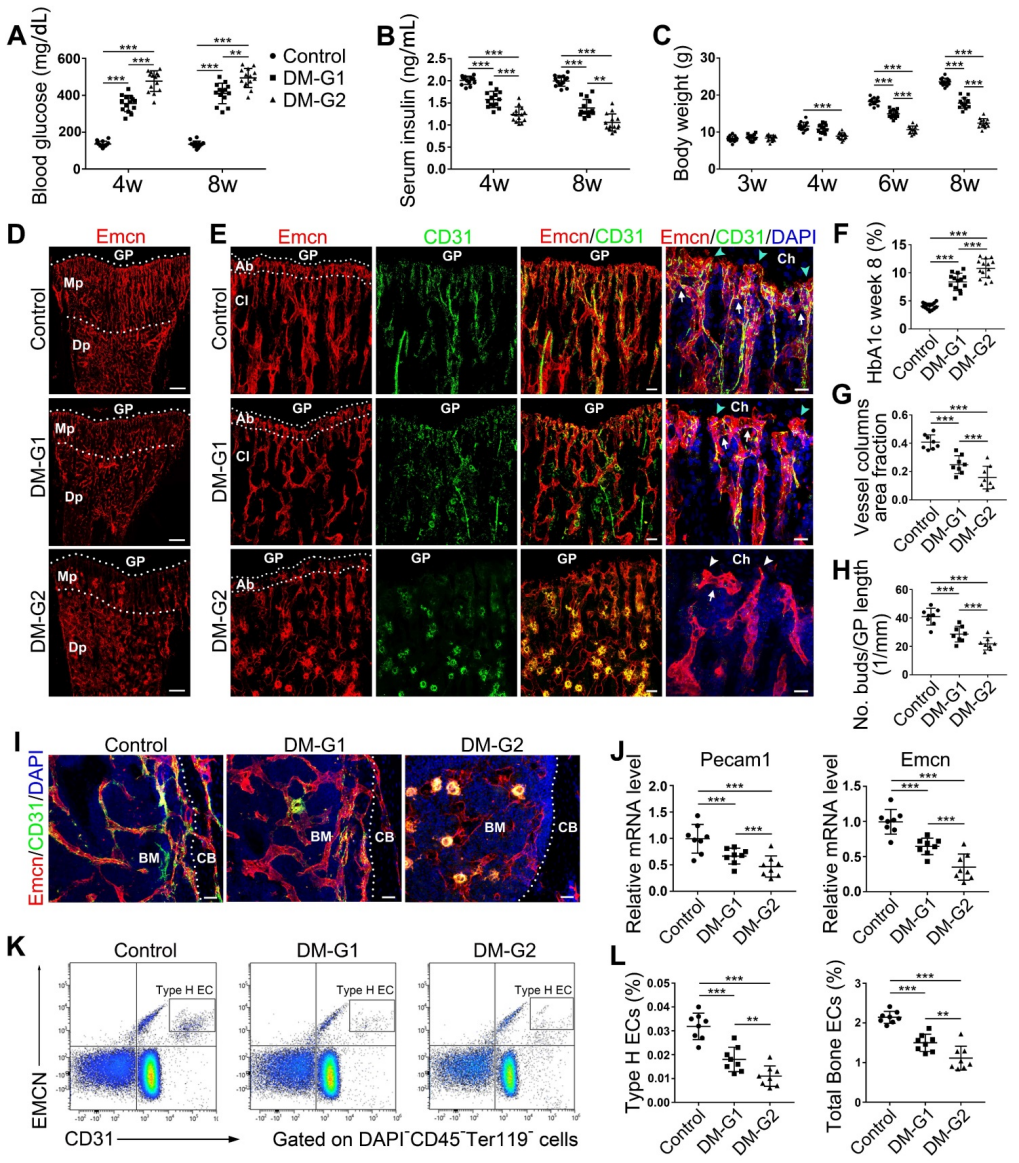
** Bone blood vessels are impaired by T1DM in a glycemic level-dependent manner.** STZ injection at two different doses led to two different levels of glucose (A), insulin (B) and HbA1c (F) in blood as well as deceased body weight gain (C), namely two degrees of diabetes which were named as DM-G1 (diabetes mellitus-glycemic level 1) group and DM-G2 (glycemic level 2) group respectively. One week after modeling (day 28), blood vessels in tibia were examined. (D) Confocal images of Emcn (endomucin), a marker of endothelial cells (ECs), on the immunostained coronal plane sections of tibia from 4-week-old mice. Dashed lines mark the boundaries between growth plate (GP) and metaphysis (Mp) as well as between the column-like vessels in metaphysis and the highly branched sinusoids in diaphysis (Dp). Scale bar: 300 μm. (E) Confocal tile scan of 4-week-old tibia showing CD31^+^ (green) and Emcn^+^ (red) ECs in metaphysis. The magnification in the last column shows the specialized angiogenesis (of type H vessels) below the growth plate. At the vascular growth front in proximity of hypertrophic chondrocytes (Ch) in the growth plate, the bud-shaped protrusions (arrowheads) grow from the distal, loop-like vascular arches (arrows). Cyan arrowheads indicate normal buds and white arrowheads mark defective buds. Dashed lines indicate the distal vascular arches and buds (Ab) in the vessel columns (Cl). Diabetes mellitus (DM) causes structural abnormality and angiogenesis inhibition of type H vessels. Scale bar: 50 μm on the left and 20 μm on the right. Histomorphometry analysis of the images represented by (E) as the area fraction of vessel columns (G) and the number of vascular buds per growth plate length (H). (I) Confocal images of the vessels near cortical bone (CB) in the diaphysis of 4-week tibia. BM, bone marrow. Scale bar: 30 μm. (J) qPCR analysis of the mRNA levels of the two markers of type H ECs, Pecam1 (CD31) and Emcn. (K) Representative flow cytometry plots showing CD31^high^Emcn^high^ ECs (type H ECs) in bone marrow cells. (L) Flow cytometric quantitation of type H and total ECs in 4-week-old tibia. Data analysis strategy in flow cytometry is diagrammed in Figure S1D. Data are present mean ± SD. (A)-(C) and (F), n = 15-16 per group; (G)-(L), n = 7-8 per group. *p < 0.05, **p < 0.01 and ***p < 0.001. One-way (F-L) or two-way (group × time, A-C) ANOVA followed by Tukey posttest analysis.

The animal experiments were carried out in strict accordance with the NIH for the Use of Laboratory Animals and the guidelines of the Fourth Military Medical University (Approval #FMMU-AEEA-20161006). Diabetes was induced in three-week-old male C57BL/6 mice to mimic early-onset T1DM in human. For modeling of T1DM, mice received an 18-hour fasting and then intraperitoneal (i.p.) injection of streptozotocin (STZ, Sigma-Aldrich) for three days. After blood glucose monitoring on 4 and 7 days post the first injection, mice with glycemic levels higher than 250 mg/dL were regarded as diabetic 16. To induce two different degrees of disease, streptozotocin was administrated at two doses, 40 mg/kg and 80 mg/kg. These two doses induced two different glycemic levels which both fulfilled the standard for T1DM (Figure 1A). The control animals were injected with only the vehicle. For experiments with drug treatments, T1DM model was induced by STZ injection at 80 mg/kg.

### Measurement of blood glucose, HbA1c and plasma insulin

To measure glycemic level, 25-μL blood samples from the tail veins of mice were collected and immediately tested by Roche glucometer (ACCU-CHEK). To measure plasma insulin level, another 100 μL blood sample from the tail vein was collected in EDTA-treated tubes and plasma was immediately isolated by centrifugation for 10 minutes at 2000× g. The plasma insulin concentrations were measured with a Mouse Insulin ELISA kit (Millipore, Cat# EZRMI-13K). HbA1c was measured with an assay kit (Crystal Chem; Cat# 80310). For the animals treated with insulin or NOX2ds-tat, blood samples were collected 4 h after drug administration.

### Drug administration

Insulin-treated animals received subcutaneous injection of insulin (Sigma-Aldrich, Cat#: I0305000) once per day in two ways, from week 4 through 8 (early-started) or from week 6 through 8 (late-started), and at two doses, a high dose (0.5 U/mouse) and a low dose (0.05 U/mouse). N-acetyl cysteine (NAC; Sigma; 10 mM), tempol (TPO; Selleckchem; 3 mM), GKT137831 (GKT, NOX1/4 inhibitor; Selleckchem; 3 mM), GSK2795039 (GSK, small-molecule NOX2 inhibitor; MedChemExpress; high dose as 4 mM and low dose as 2 mM) and VAS2870 (VAS, small-molecule NOX2 inhibitor; Abcam; 3 mM) were given in drinking water as the only drinking source for mice every two days from week 4 through 8 17-19. NOX2ds-tat (NOX2ds, short-peptide NOX2 inhibitor; AnaSpec; 5 mg/kg) were i.p. injected once every other day from week 4 through 8 20.

### Bone immunohistochemistry

Freshly dissected mice bone tissues were immediately fixed in ice-cold 4% paraformaldehyde solution for 8 h, and decalcified in 0.5 M EDTA (pH 7.4) at 4 ℃ for 72 h. The decalcified bones were then incubated in 20% sucrose and 2% polyvinylpyrrolidone (PVP) solution for 24 h. Finally, the tissues were embedded in 8% gelatin (porcine; Sigma, G2500) in presence of 20% sucrose and 2% PVP (Sigma, PVP360) and cryosectioned into 40-μm thick longitudinally oriented tibia sections in a cryostat (Leica CM1800). For immunostaining, bone sections were permeabilized for 15 min in 0.3% Triton X-100, blocked in 5% donkey serum at room temperature (RT) for 30 min and then probed with the primary antibodies diluted in 5% donkey serum in PBS: rabbit-anti CD31 (Abcam, ab28364; 1:50), goat-anti endomucin (Emcn; R&D system, AF4666; 1:100), rabbit-anti Osterix (Abcam, ab22552; 1:200) and rat-anti Ki-67 (Invitrogen, 14-5698-82; 1:200) overnight at 4 ℃. After being washed with PBS for three times, sections were incubated with secondary antibodies to corresponding species conjugated with Alexa 488, 594 or 647 (Millipore; 1:500) at RT for 1.5 h while avoiding light 11, 14. Nuclei were counterstained with 40, 60-diamidino-2-phenylindole (DAPI, Sigma). After being washed thoroughly in PBS, air-dried and coverslipped, the sections were observed under a confocal laser microscope (FV-1000, Olympus). Tartrate-resistant acid phosphatase (TRAP) staining of bone sections were performed using a staining kit (Sigma-Aldrich). Histomorphometric analysis was conducted in four random visual fields of metaphysis per tibia section in five non-adjacent sections per mouse, with images quantitatively analyzed by ImageJ software.

### Flow cytometry

For the analysis or sorting of CD31^high^Emcn^high^ cells (type H ECs) and total ECs in bone, we collected tibiae after euthanization of the mice, removed epiphysis, muscles and periosteum around the bone. To remove epiphysis, the epiphysis and diaphysis of tibia were respectively held and carefully moved using a tweezer. Then the bone was crushed in ice-cold PBS and digested by collagenase (Sigma, 1 mg/mL) at 37 ℃ for 20 min to obtain single-cell suspensions. After filtration and washing, equal numbers of cells per mouse were incubated with goat-anti endomucin (R&D Systems, AF4666, 1:50) and biotin-coupled CD45 (BD, 553077, 1:100) and Ter119 (BD, 559971, 1:100) antibodies for 45 min at 4 ℃. After washing, cells were stained with anti-goat APC (R&D Systems, F0108, 1:200), PE-conjugated CD31 (BioLegend, 102508, 1:100) and Streptavidin-PE/Cy7 (BioLegend, 405206, 1:200) antibodies for 30 min at 4 ℃. After washing, cells were resuspended in PBS with 1 µg/mL DAPI (live/dead exclusion), acquired on a FACS Canto flow cytometer (BD Biosciences) and analyzed using FlowJo software (Tree Star). Total bone ECs were quantified as CD31^+^CD45^-^Ter119^-^ cells 11. For demarcating and sorting type H ECs, first standard quadrant gates were set and subsequently quadrant 2 gates were arbitrarily set to differentiate CD31^high^Emcn^high^ cells from the total double positive cells 11. FITC-conjugated Ki-67 antibody (BioLegend, 652410, 1:200) was used to detect cell proliferation. The strategy to sort type H and total endothelial cells is diagrammed in Figure S1D. Antibody concentrations were selected according to the manufacturers' instructions and our preliminary experiments.

To determine the level of reactive oxygen species (ROS) in bone ECs, the single-cell suspensions of tibia were first incubated with biotin-coupled CD45 and Ter119 antibodies for 45 min at 4 ℃. After washing, cells were stained with Streptavidin-PE/Cy7 and CD31-PE antibodies as well as 10 μmol/L 2',7'-dichlorodihydrofluorescein diacetate (DCFH-DA, Sigma) for 30 min at 4 ℃. The general ROS levels in bone ECs were determined as the intensity of DCF fluorescence in CD31^+^ CD45^-^ Ter119^-^ cells 21. To determine intracellular ROS levels in experiments *in vitro*, cells cultured on plates were digested, washed with PBS and incubated with 10 μmol/L DCFH-DA at 37 ℃ for 30 min. After being washed three times with PBS, the levels of DCF fluorescence in ECs were quantified using flow cytometry.

### Quantitative Real time-PCR (qPCR)

Pure EC fractions were flow-sorted directly into the lysis buffer of RNeasy Mini Kit (Qiagen). qPCR analysis was performed using standard procedures as previously described 11, 22 to determine the mRNA levels of Nog, Pdgfa, Tgfb1, Fgf1, Wnt3a, Bmp2, Hif1a, Nox1, Nox2 and Nox4 in ECs. For the analysis of Pecam1, Emcn, Bgalp, Ibsp, Ctsk and Oscar expressions in whole-bone samples, freshly dissected bones were immediately snap-frozen in liquid nitrogen and crushed. The pulverized bone shafts were lysed in lysis buffer for 15 min before proceeding for RNA isolation, cDNA preparation and qPCR analysis as described above. All the gene expressions were normalized to endogenous Actb. The primer sequences were listed in Table S2.

### Micro-CT analysis of bone structure

Tibia were dissected from mice, fixed overnight in 10% formalin and analysed by micro-CT (eXplore Locus SP Pre-Clinical Specimen, GE Healthcare). The samples were scanned in air, at a tube potential of 80 kVp, a tube current of 80 μA and an isotropic voxel size of 7 μm. The image reconstruction and data analysis were done by the system software (MicroView, v.2.1, GE healthcare) with a Gaussian filter (sigma = 0.8, support = 1). Trabecular bone within the metaphysis was segmented from the cortex using a semi-automated contouring algorithm by manually drawing contours every 0.5 mm in the axial plane and the volume of interest (VOI) was selected from about 5% of the total tibia length below the growth plate extending distally for another 10% of the total tibia length. For cortical bone, the VOI was selected by a software-original automated contouring algorithm as a region with X-ray signal value higher than a manually set threshold and defined as a 0.5-mm-thick transverse region of the diaphysis 5 mm below the growth plate. Analysis parameters were reported according to the guideline from American Society for Bone and Mineral Research 23.

### Examination of dynamic bone formation

Mice received subcutaneous injection of calcein (Sigma, 20 mg/kg, at week 4) and Alizarin-complexone (Sigma, 25 mg/kg, at week 6) dissolved in PBS. After micro-CT scanning, bone specimens were dehydrated in a graded series of ethanol (70%-100%) and embedded in methylmethacrylate (Sigma) solution that polymerized within 7 days at room temperature. Thin undecalcified sections (50 μm in thickness) were prepared in an interlocked diamond saw (Leica Microtome, Wetzlar, Germany) to observe and obtain images of the double labeling (green and red) under a fluorescence microscope 24, with images analyzed by Image J software. Four randomly selected visual fields in the metaphysis of tibia were measured to test trabecular bone formation, with three slices examined for each animal.

### Biomechanical testing

To measure the biomechanical properties of tibia, a mechanical-testing machine (MTS 858 Mini Bionix II; MTS Systems Corp., Minneapolis, MN, USA) was used to run 3-point bending test. The length span between 2 support points was 60% of the total bone length. Each bone was loaded at a constant speed of 1 mm/min until failure. The load-deformation curves were generated and analyzed by TestStar II software. The force and displacement data were recorded until the specimen was broken. The following biomechanical parameters were determined by the load-deformation curve: ultimate load (N; the load at the maximum failure point) and stiffness (N/mm; the slope of the linear region).

### Isolation and culture of primary bone-specific endothelial cells (ECs)

For the isolation of mouse bone-specific ECs, bone marrow from tibia and femurs of C57BL/6 mice (male, 4-week-old) were collected in sterile Ca^2+^ and Mg^2+^ free PBS 25. After crushing the bone, the mixture was digested with collagenase (Sigma) and filtered by a strainer to obtain single-cell suspension. ECs were then sorted by Magnetic Activated Cell Sorting (MACS) using endomucin antibody (Santa Cruz) and Dynabeads coated with sheep anti-rat antibody (Invitrogen). Sorted ECs were then plated on fibronectin-coated dishes and cultured in endothelial cell growth medium (EBM-2, Lonza) with EGM-2 SingleQuots supplement (CC-4176, Lonza) at 37 ℃ with 5% CO_2_ in a humidified atmosphere. At first passage, cells were again MACS sorted with endomucin antibody and plated for culture. Cells were fed every third day and passaged upon confluence. ECs were cultured in three different conditions: normal control, high glucose (HG, 400 mg/dL) and HG + tempol (TPO, 2.5 mM). Cells behaviors were evaluated after 4 days.

### Cell adhesion and viability

After fixation by 4% paraformaldehyde and washing by PBS, cellular actin filaments and nucleus were stained respectively with Rhodamine-phalloidin (Molecular Probes) and DAPI (Sigma). Fluorescent images were obtained by a confocal microscope. The cell density was measured using the Image J software. Five different substrate fields were measured per sample, and three separate samples were measured in each group. Cell viability was assessed by methylthiazol tetrazolium (MTT) test (Beyotime Biotechnology, Shanghai, China) and expressed as the absorbance per gram of sample.

### Statistical analysis

Results are expressed as mean ± SD. GraphPad Prism 7 was used to compare two groups by two-tailed Student t-test or to compare more than two groups by one-way or two-way (group × time) ANOVA with Tukey posttest multiple comparison analysis. A *p* < 0.05 was considered statistically significant.

## Results

### T1DM impaired bone blood vessels in a glycemic level-dependent manner

Most T1DM patients have disease onset at a young age 26. Clinical data showed that children and adolescents with T1DM had abnormal bone quality 3, 27, suggesting that T1DM may impair bone growth and increase bone fragility 1, 3. Although diabetes in children and adolescents has been increasing at an alarming rate 28, significant gaps still exist in our understanding of diabetes in juveniles. In the present study, we established an early-onset T1DM mouse model by post-fasting injection of STZ in week 3 at two different doses, 40 and 80 mg/kg, leading to two different levels of blood glucose, HbA1c, insulin, and body weight gain in diabetic animals (Figure 1A-C and F). The subgroup of T1DM mice with lower STZ dose had relatively lower glycemic levels (diabetes mellitus-glycemic level 1, DM-G1) and the other T1DM subgroup with higher STZ dose had higher glycemic levels (DM-glycemic level 2, DM-G2). Immunofluorescence staining of the tibia sections on day 28 showed obvious morphological changes of blood vessels in the bones of DM animals compared with control animals (Figure 1D-E), which became severe with the rise of the glycemic level, including abnormal and sparse vascular network. Additionally, the vascular columns under the growth plate (GP), which regulate trabecular bone metabolism in the metaphysis (Mp), became shorter and fewer (Figures 1D, G and S1A). Magnification of metaphysis (Figure 1E) showed that the number of Emcn^+^CD31^+^ type H vessels adjacent to the growth plate was decreased in DM groups, with many aberrant circular immunostaining-positive structures in the DM-G2 group. In the tibia of the control group, the specialized angiogenesis pattern in the bone was observed in the metaphysis, with the bud-shaped protrusions (arrowheads) growing out of the distal, loop-like arches (arrows) at the vascular growth front in the proximity of the hypertrophic chondrocytes (Ch) in the growth plate. In DM groups, the vascular arches showed altered structures and significantly fewer angiogenesis buds (Figure 1H). In diaphysis (Dp), type H vessels near endosteum were reduced in DM groups compared with the control group (Figures 1I and S1B). qPCR analysis showed that the Pecam1 (CD31) and Emcn mRNA levels were significantly lower in DM tibia, with a remarkable difference between the two DM subgroups (Figure 1J). Flow cytometry further showed significantly fewer type H (Figure 1K-L) and type L (Figure S1C) ECs as well as the total ECs in the tibia in DM groups than the control group. Correlation analysis showed that the ratio of type H ECs in tibia was negatively related to the blood glucose level in DM groups (Figure S2). The proliferation of both type H and total ECs in 4-week-old tibia was remarkably inhibited by T1DM (Figure S1E-F).

### T1DM inhibited angiogenesis-osteogenesis coupling

**Figure 2:**
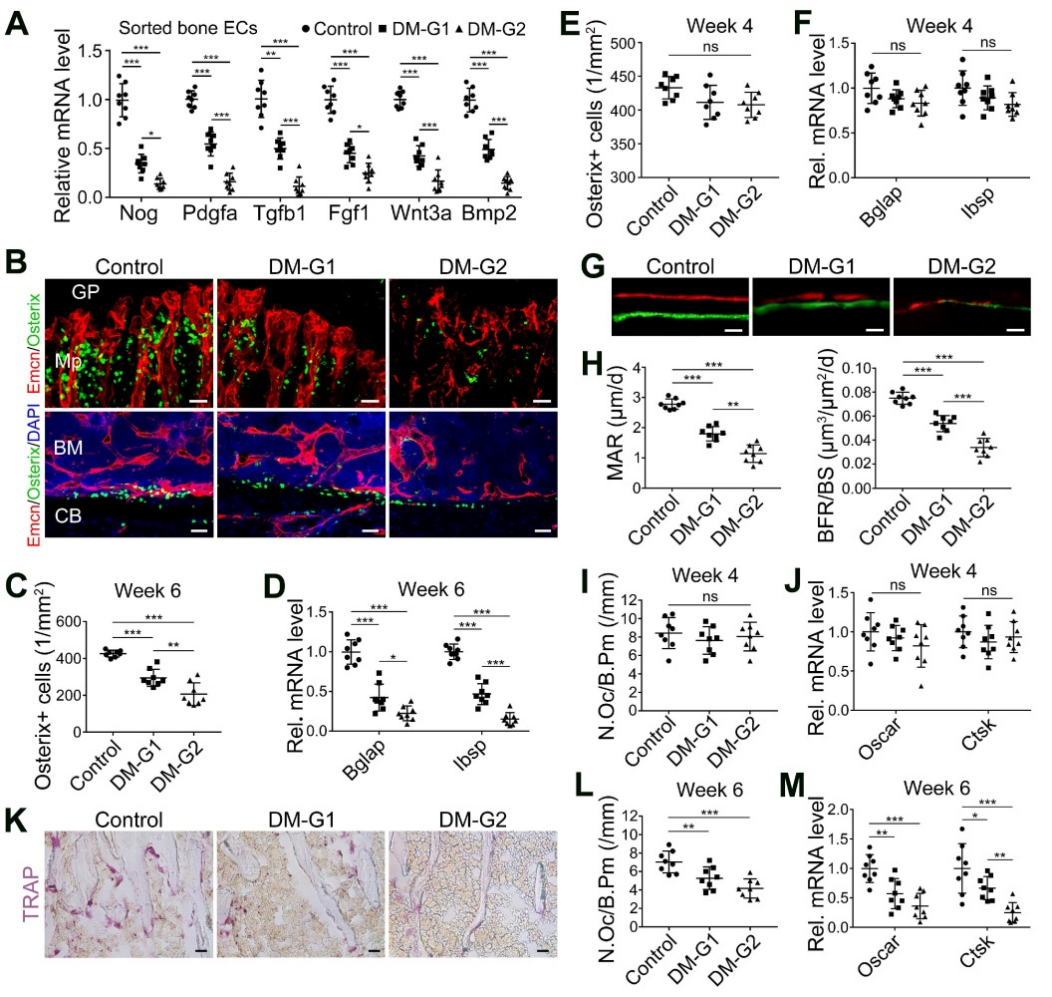
** T1DM led to the uncoupling of angiogenesis and osteogenesis in a glycemic level-dependent manner and hindered both bone formation and resorption.** (A) qPCR for the expression fold changes of known pro-osteogenic factors (normalized to Actb) in sorted bone ECs from 4-week-old tibia, relative to control group. (B) Confocal images show the Osterix^+^ (green) osteoprogenitors adjacent to Emcn^+^ (red) blood vessels in metaphysis (Mp, above) and diaphysis (below). GP, growth plate; CB, cortical bone; BM, bone marrow. Scale bar: 20 μm on the above and 30 μm on the below. Histomorphometric quantitation of Osterix^+^ cells in the metaphysis of immunostained tibia sections at week 4 (E) and week 6 (C). qPCR analysis of the mRNA expressions of two markers for mature osteoblasts, Bglap (bone gamma-carboxyglutamate protein) and Ibsp (integrin-binding sialoprotein), relative to vehicle control group at week 4 (F) and week 6 (D). Dynamic new bone formation was assessed by subcutaneous injection of calcein (green, at 4 weeks) and alizarin complexone (red, at 6 weeks) to label the mineralization in bone (G, microscopy images of tibia sections) as well as histomorphometric quantifications (H) of mineral apposition rate (MAR) and bone formation rate per bone surface (BFR/BS). (K) Images of TRAP-stained tibia sections. Histomorphometric analysis of the number of osteoclasts per bone perimeter (N.Oc/B.Pm.) at week 4 (I) and week 6 (L). Relative mRNA levels of the markers for osteoclast differentiation (Oscar, osteoclast associated receptor) and function (Ctsk, cathepsin K) at week 4 (J) and week 6 (M). TRAP, tartrate-resistant acid phosphatase. Scale bars: 20 µm in (G) and (K). n = 7-8 per group. *p < 0.05, **p < 0.01 and ***p < 0.001. One-way ANOVA followed by Tukey posttest analysis.

The coupling between angiogenesis and osteogenesis in the bone is mediated by the dialogue between vascular cells and osteoblastic cell lineage 11, 14, 29, 30. Type H ECs regulate osteoblastic cells by paracrine key cytokines crucial for osteogenesis, such as noggin, BMP-2, and TGF-β, known as angiocrine functions 11, 13. The expression of these key cytokines was significantly reduced in the diabetic tibia following the vascular impairment in bone in week 4, as determined by qPCR analysis (Figure 2A). Histomorphometric analysis showed that DM led to a significant reduction of Osterix^+^ osteoprogenitors in the metaphysis and near the endosteum in diaphysis, accompanied by decreased expression of mature osteoblast markers, Bglap and Ibsp, at week 6 (Figure 2B-D) but not at week 4 (Figure 2E-F). Fluorochrome labeling of bone formation by calcein (green, at 4 w) and alizarin complexone (red, at 6 w) showed that the osteogenesis in bone development was inhibited by T1DM (Figure 2G), with significantly reduced mineral apposition rate (MAR) and bone formation rate per bone surface (BFR/BS, Figure 2H). Histomorphometric quantification and qPCR analysis also revealed that the number of TRAP^+^ osteoclasts and the mRNA levels of osteoclast differentiation (Oscar, osteoclast associated receptor) and function (Ctsk, cathepsin K) significantly decreased in diabetic tibia at week 6 (Figure 2K-M), but not at week 4 (Figure 2I-J).

### T1DM led to compromised bone mass and decreased bone strength

**Figure 3:**
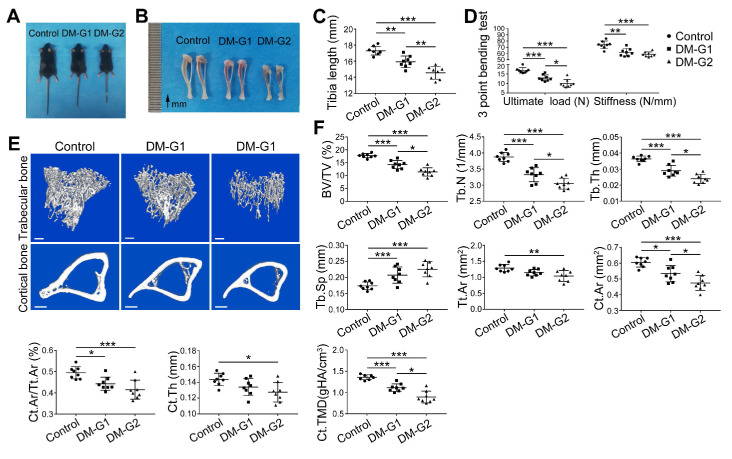
** T1DM induced compromised quality of bone mass at 8 weeks.** Comparisons of body size (A), tibia appearance (B) and tibia length (C). D) 3-pointing bending test evaluates the mechanical properties of mice tibia: ultimate load, the load at the maximum failure point; stiffness, the slope of the linear region of the load-deformation curve. (E) Representative images from micro-CT scanning show the trabecular and cortical bone mass in mice tibia: above, 3-dimensional (3D) reconstruction of the trabecular bone in proximal tibia; below, cross-section images of the middle diaphysis of tibia. Scale bars: 300 µm. (F) Quality parameters of the trabecular bone in proximal tibia metaphysis and the cortical bone in middle diaphysis from micro-CT analysis: BV/TV, trabecular bone volume fraction; Tb.N, trabecular number; Tb.Th, trabecular thickness; Tb.Sp, trabecular separation; Tt.Ar, Total cross-sectional area inside the periosteal envelope; Ct.Ar, cortical bone area; Ct.Ar/Tt.Ar, cortical bone area fraction; Ct.Th, average cortical thickness; Ct.TMD, Cortical tissue mineral density. n = 7-8 per group. *p < 0.05, **p < 0.01 and ***p < 0.001. One-way ANOVA followed by Tukey posttest analysis.

At week 8, compared with the control group, DM animals had a significantly smaller size of the body and long bone (Figure 3A-C). Micro-CT analysis of 8-week-old tibia showed that the trabecular bone in the tibia of DM groups was significantly reduced (Figure 3E-F), indicated by decreased trabecular bone volume fraction (BV/TV), trabecular number (Tb.N) and trabecular thickness (Tb.Th) as well as increased trabecular separation (Tb.Sp). For the cortical bone, diabetic tibia showed a significant reduction in total cross-sectional area of the middle diaphysis (Tt.Ar), cortical bone area (Ct.Ar), cortical area fraction (Ct.Ar/Tt.Ar), average cortical thickness (Ct.Th), and cortical tissue mineral density (Ct.TMD). Mechanical properties of the tibia were assessed by the 3-point bending test (Figure 3D). Compared with the control group, the ultimate load and stiffness of the bone were significantly decreased in DM groups, with the ultimate load considerably lower in the DM-G2 group than in the DM-G1 group. In most bone mass indexes, the DM-G2 group showed significantly severer pathological changes than the DM-G1 group.

### Early-started, but not late-started, sufficient insulin therapy attenuated the T1DM-induced bone vascular injury and osteogenesis inhibition

**Figure 4:**
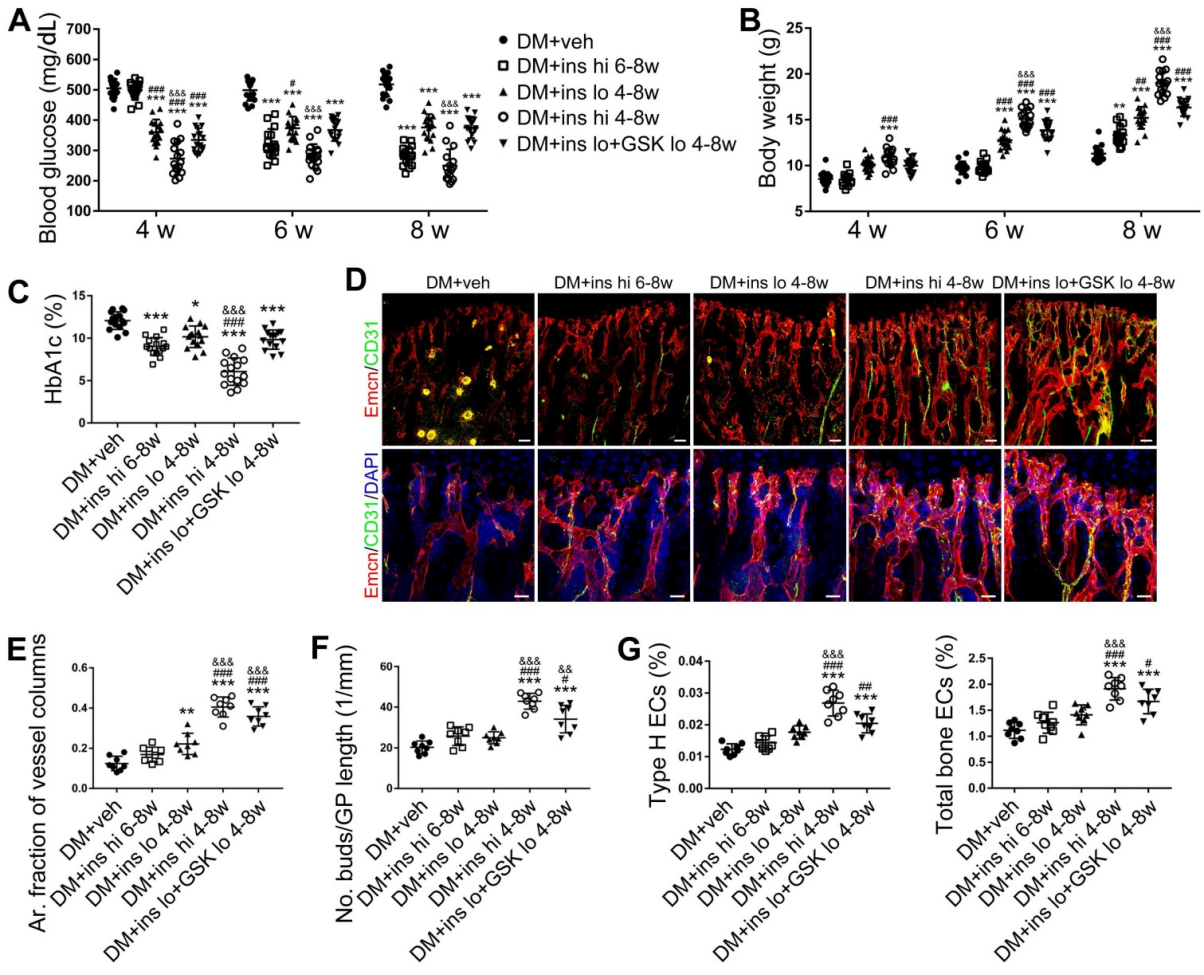
** The effects of insulin therapy on the bone blood vessels of T1DM mice.** Drug therapy with high dose (hi, 0.5 U/mouse) or low dose (lo, 0.05 U/mouse) of insulin (ins) or the combination of insulin and a relatively low dose of GSK2795039 (GSK, a NOX2 inhibitor, 2 mM) was conducted in two time courses: started immediately after the confirmation of DM and once per day in week 4 to 8 (4-8w); started at week 6 and performed once per day in week 6 to 8 (6-8w). The levels of blood glucose (A) and body weight (B) were examined at week 4, 6 and 8, with HbA1c (C) tested at week 8. (D) Confocal images of immunostained tibia sections show CD31^+^ (green) and Emcn^+^ (red) type H vessels in the metaphysis. Scale bar: 50 μm on the above and 20 μm on the below. Histomorphometric quantitation as the area fraction of vessel columns (E) and the number of vascular buds per growth plate length (F). (G) Flow cytometry quantification of type H ECs and total ECs in 8-week-old tibia. For drug treatments, T1DM model was induced by STZ injection at 80 mg/kg (namely DM-G2). (A)-(C), n = 15-16 per group; (E)-(G), n = 6-8 per group. *p < 0.05, **p < 0.01 and ***p < 0.001 *vs*. DM+veh group; ^#^*p* < 0.05, ^##^*p* < 0.01 and ^###^*p* < 0.001 *vs*. DM+ins hi 6-8w group; ^&^*p* < 0.05, ^&&^*p* < 0.01 and ^&&&^*p* < 0.001 *vs*. DM+ins lo 4-8w group. One-way or two-way ANOVA followed by Tukey posttest analysis.

**Figure 5:**
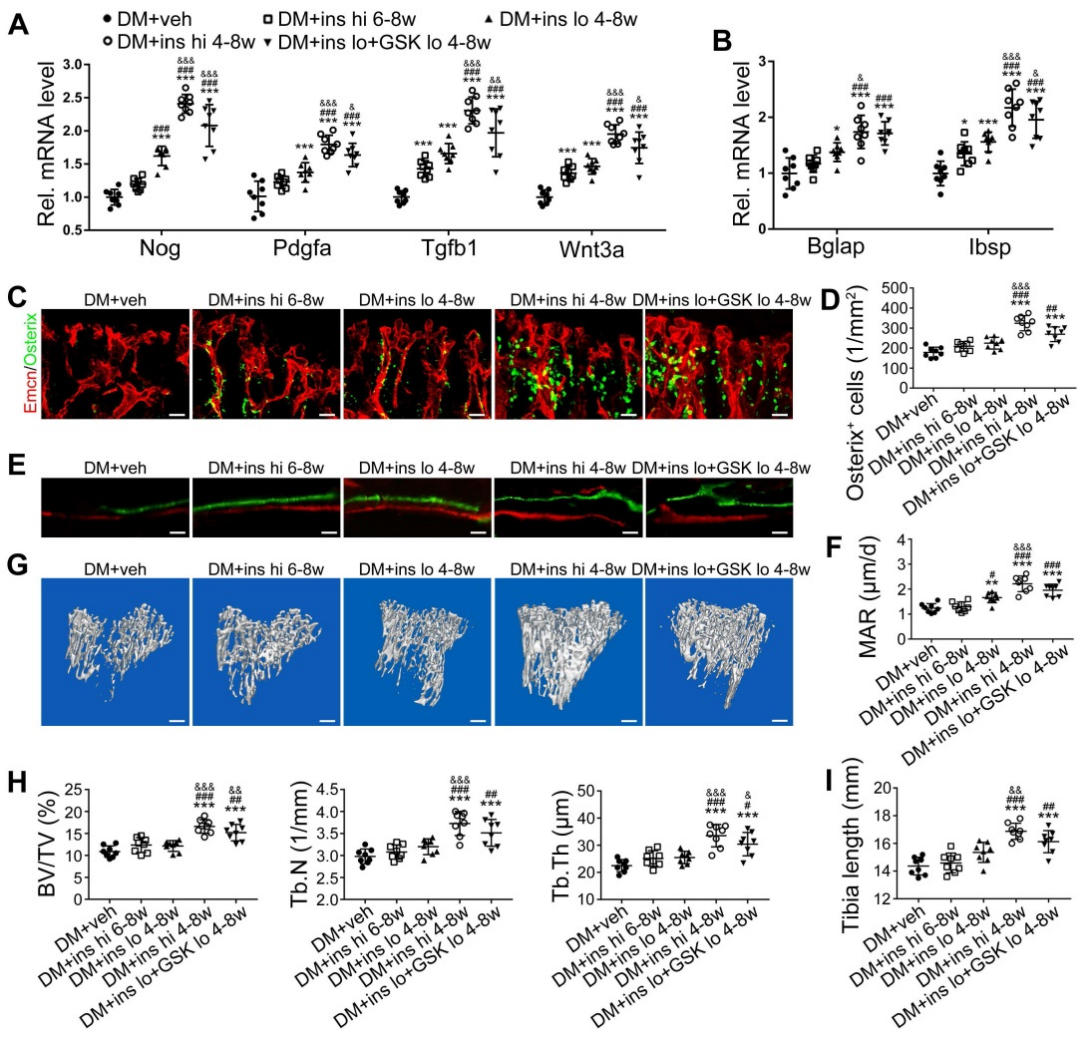
** The effects of insulin therapy on the angiogenesis-osteogenesis coupling and bone mass of T1DM mice.** (A) qPCR analysis of the relative mRNA expressions of key cytokines mediating the coupling of angiogenesis and osteogenesis in 8-week-old tibia. The activities of osteoblastic cell lineage were evaluated by qPCR analysis of osteoblast markers (B) and histomorphological analysis of Osterix^+^ (green) osteoprogenitors around Emcn^+^ vessels (C and D). Analysis of new bone formation by fluorochrome labeling (E) of calcium deposition in tibia by subcutaneous injection of calcein (green) and alizarin complexone (red) and quantitation of mineral apposition rate (MAR) between week 4 and 6 (F). Micro-CT 3D reconstruction images (G) and quantitative parameters (H) of trabecular bone in 8-week-old tibia. (I) Tibia length at week 8. Scale bar: 20 μm in (C) and (E); 300 μm in (G). n = 6-8 per group. *p < 0.05, **p < 0.01 and ***p < 0.001 *vs*. DM+veh group; ^#^*p* < 0.05, ^##^*p* < 0.01 and ^###^*p* < 0.001 *vs*. DM+ins hi 6-8w group; ^&^*p* < 0.05, ^&&^*p* < 0.01 and ^&&&^*p* < 0.001 *vs*. DM+ins lo 4-8w group. One-way or two-way ANOVA followed by Tukey posttest analysis.

Since T1DM is an insulin-deficient disease, insulin replacement therapy has been the standard treatment. We evaluated insulin effects on bone blood vessels and bone mass by daily intraperitoneal (i.p.) injections of insulin in the early treatment starting from week 4 (ins 4-8w) and late treatment from week 6 (ins 6-8w) with high, 0.5 U/mouse and low, 0.05 U/mouse doses. Insulin treatments starting at the two time points both effectively decreased the glycemic level (Figure 4A) and increased body weight gain (Figure 4B) in DM mice but displayed significant differences between the two doses. Although the late-started insulin therapy from week 6 through 8 increased mRNA levels of Tgfb-1, Wnt3a and Ibsp, it did not induce observable changes in blood vessels, osteoblastic cells, or osteogenesis in the tibia of DM mice (Figure 4, DM + ins hi 6-8w group). However, the insulin treatment at a sufficient dose started immediately after the confirmation of diabetes modeling at week 4 (DM + ins hi 4-8 w group) led to a significant increase in both the total ECs and type H ECs (Figure 4D-G), cytokines mediating the coupling of angiogenesis and osteogenesis (Figure 5A), number and function of osteoblastic cells (Figure 5B-D), bone formation (Figure 5E-F), osteoclast activity (Figure S3A-C), bone mass quality (Figure 5G-H and Figure S4A), and total length (Figure 5I) of the tibia of DM mice. However, only limited effects were observed when insulin therapy started at week 4 but with a relatively low dose (DM + ins lo 4-8w group).

### Anti-oxidants effectively ameliorated the T1DM-induced impairment of bone blood vessels and bone formation

**Figure 6:**
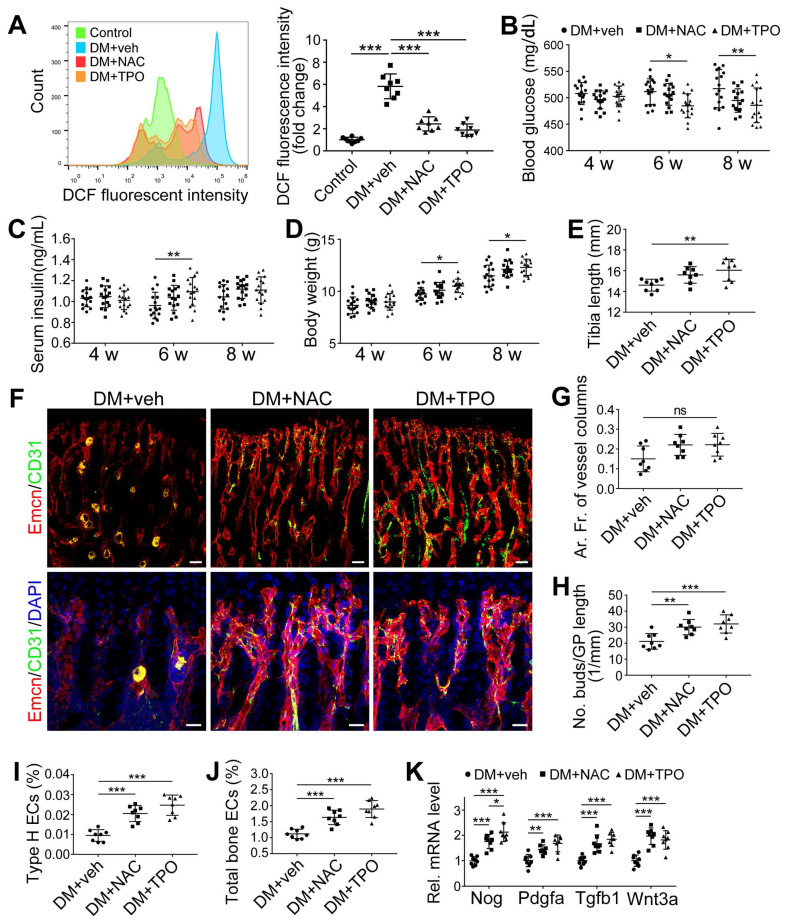
** The effects of anti-oxidant treatments on the bone blood vessels of T1DM mice.** T1D mice received intraperitoneal injection of an anti-oxidant, N-acetyl cysteine (NAC) or tempol (TPO), once every other day from week 4 through 8. (A) Flow cytometry analysis of the general ROS levels in the bone ECs from 8-week-old tibia by detecting intracellular DCF fluorescence. The levels of blood glucose (B), insulin (C) and body weight (D). (E) Tibia length at week 8. (F) Confocal images of CD31 (green) and Emcn (red) immunostained tibia sections show type H vessels in the tibia metaphysis at week 8. Scale bar: 50 μm on the above and 20 μm on the below. Histomorphometric quantitation of the area fraction of vessel columns (G) and the number of vascular buds per growth plate length (H). Flow cytometry quantification of type H ECs (I) and total ECs (J) in 8-week-old tibia. (K) qPCR analysis of the representative cytokines mediating the coupling of angiogenesis and osteogenesis in 8-week-old tibia. (B)-(D), n = 15-16 per group; other panels, n = 7-8 per group. *p < 0.05, **p < 0.01 and ***p < 0.001. One-way or two-way ANOVA followed by Tukey posttest analysis.

**Figure 7:**
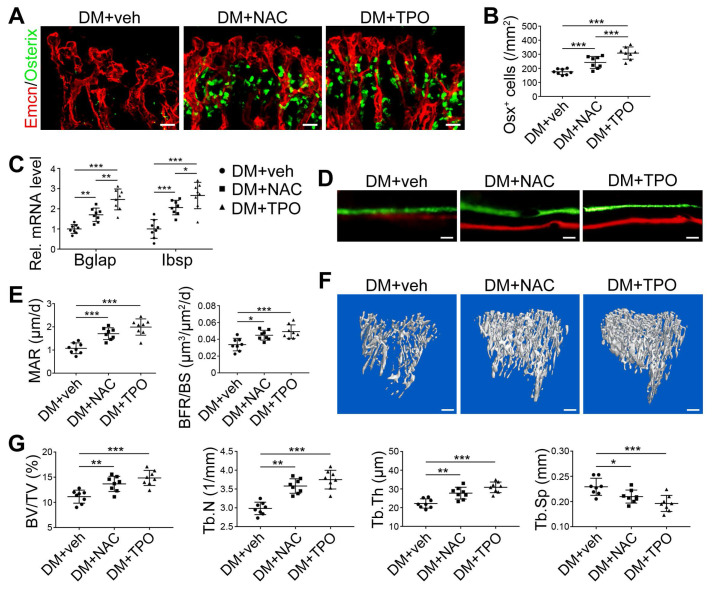
** The effects of anti-oxidant treatments on the angiogenesis-osteogenesis coupling and bone mass of T1DM mice.** The activities of osteoblastic lineage were evaluated by histomorphological analysis of Osterix^+^ (green) osteoprogenitors around Emcn^+^ vessels (A and B) and qPCR analysis of osteoblast markers (C). New bone formation was analyzed by fluorochrome labeling of bone mineralization (D) as well as histomorphometric analysis (E) of mineral apposition rate (MAR) and bone formation rate per bone surface (BFR/BS) between week 4 and 6. Micro-CT 3D reconstruction images (F) and quantitative parameters of trabecular bone (G) in 8-week-old tibia. Scale bar: 20 μm in (A) and (D); 300 μm in (F). n = 7-8 per group. *p < 0.05, **p < 0.01 and ***p < 0.001. One-way or two-way ANOVA followed by Tukey posttest analysis.

Overproduction of reactive oxygen species (ROS) and the subsequent oxidative stress have been demonstrated to play a critical role in vascular lesions and diabetic vascular complications 31. ROS has been identified as a key regulator of the bone micro-environment 32, 33. We explored the role of oxidative stress in the diabetes-induced damage of bony vasculature by evaluating the ROS level in bone ECs. Flow cytometry analysis of intracellular DCF fluorescence showed that the EC ROS level in diabetic tibia was significantly higher than in the control tibia (Figure 6A and Figure S5A). When diabetic animals received i.p. injection of an anti-oxidant, N-acetyl cysteine (NAC) or tempol (TPO) once every other day from week 4 through 8, the ROS level in tibia ECs was effectively reduced (Figure 6A). Diabetic mice treated with TPO showed significantly reduced glycemic levels and increased body weight at weeks 6 and 8 (Figure 6B, C, and D), while those effects were insignificant after NAC treatment. Both NAC and TPO ameliorated the diabetes-induced injury of bone ECs (Figure 6F-J), uncoupling of angiogenesis and osteogenesis (Figure 6K and Figure 7A-C), and bone formation inhibition in the tibia (Figure 7D-G and Figure S4B). The tibia length also increased in TPO-treated diabetic mice (Figure 6E).

To investigate the direct effects of high glucose and antioxidants on the vascular endothelium in the bone, we isolated bone-specific ECs from the long bone and cultured them under different conditions. Compared with the normal control condition, high glucose (400 mg/dL) resulted in reduced cell proliferation (Figure S6A-B) and viability (Figure S6C) and a significantly increased ROS level in ECs (Figure S6D-E). Hypoxia inducible factor-1α (Hif-1α), a master regulator of the expression of a large number of genes that play critical roles in vascular activities, including the angiogenesis of type H vessels 11, 34, was sensitive to oxygen and could be inhibited by ROS 35. In the high glucose environment, the expressions of Hif-1α and osteogenesis-regulating cytokines were downregulated in bone ECs (Figure S6F), while these high glucose-induced cell dysfunctions were significantly ameliorated by the antioxidant, TPO (Figure S6).

### Inhibition of NOX2, rather than NOX1, attenuated T1DM-induced bone vascular lesions

**Figure 8:**
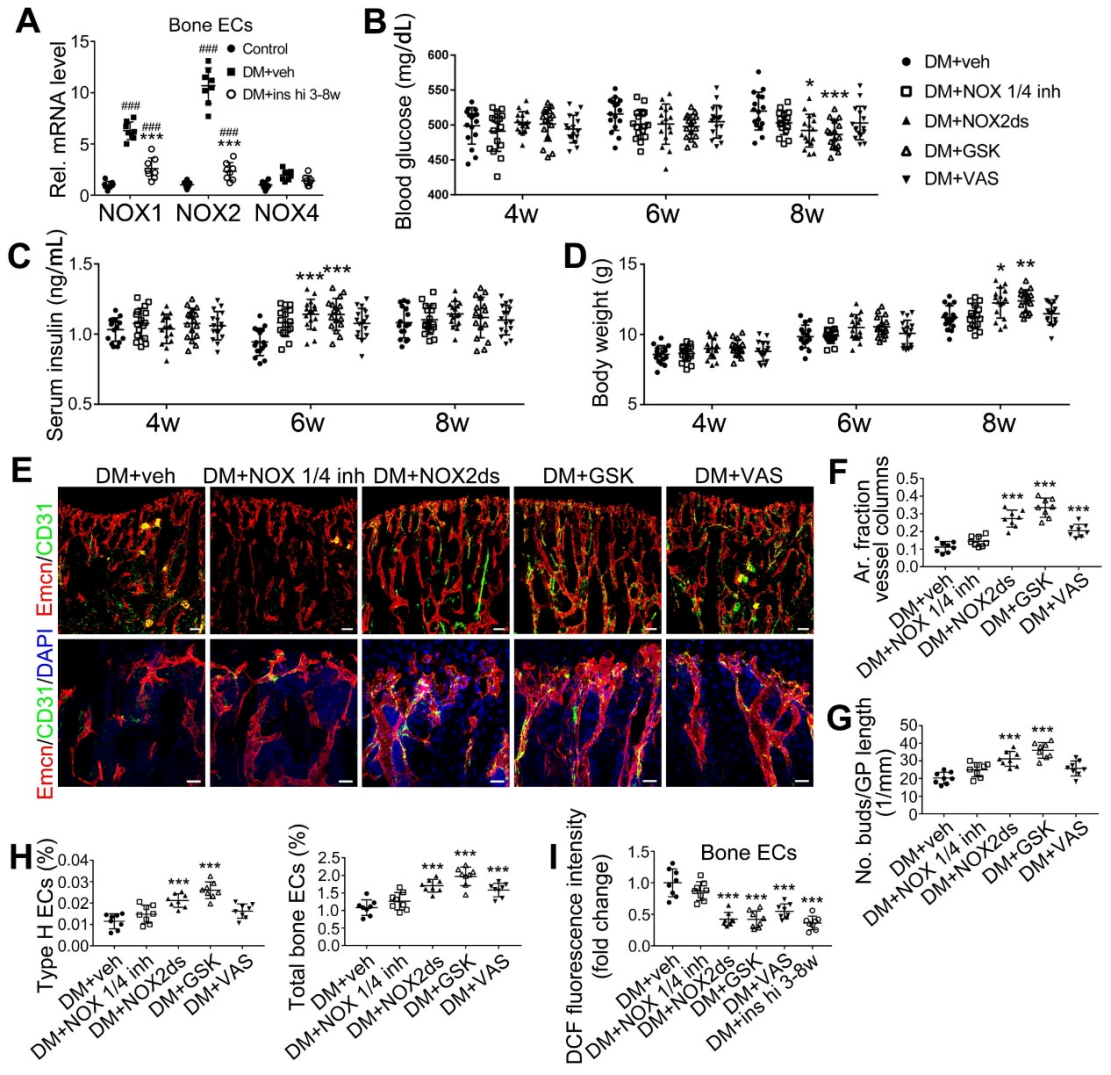
** NOX2 contributes to the oxidative stress of bone ECs and the damage of angiogenesis-osteogenesis coupling**. (A) qPCR analysis of the relative mRNA levels of three critical NOXs, involved in endothelial oxidative injuries, in the ECs in tibia metaphysis. T1DM mice were intraperitoneally injected with GKT137831 (NOX1/4 inhibitor) or one of the two NOX2 inhibitors, NOX2ds-tat (NOX2ds) and GSK2795039 (GSK), or VAS2870 (VAS, a pan-NOX inhibitor) once every two days from week 4 through 8. The levels of blood glucose (B), insulin (C) and body weight (D) during treatments were tested. (E) Confocal images of immunostained tibia sections show the CD31^+^Emcn^+^ type H vessels in the metaphysis at week 8. Scale bar: 50 μm on the above and 20 μm on the below. (F and G) Histomorphometric quantitation of the vessels in proximal tibia metaphysis. Flow cytometry quantification of type H ECs and total ECs in 8-week-old tibia (H). (I) Flow cytometry analysis of intracellular DCF fluorescence shows the ROS levels in the ECs in 8-week-old tibia. (B)-(D), n = 15-16 per group; other panels, n = 7-8 per group. ^###^*p* < 0.001* vs*. Control group; *p < 0.05, **p < 0.01 and ***p < 0.001 *vs*. DM+veh group. One-way or two-way ANOVA followed by Tukey posttest analysis.

**Figure 9:**
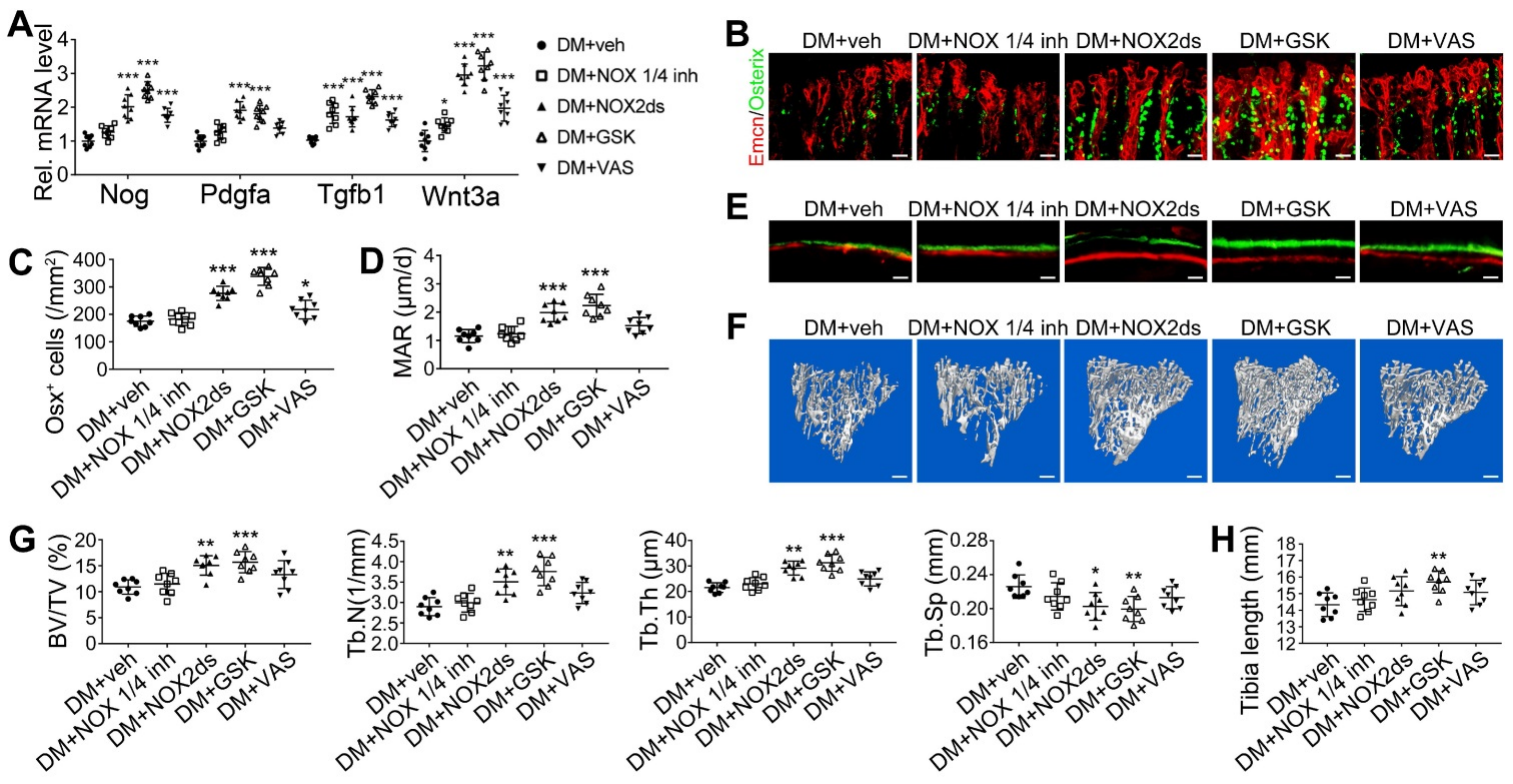
** The effects of NOX inhibitors on the angiogenesis-osteogenesis coupling and bone mass of T1DM mice.** (A) qPCR analysis of representative key cytokines mediating the coupling of angiogenesis and osteogenesis in 8-week-old tibia. Osterix^+^ (green) osteoprogenitors around vessels in the metaphysis (B) were semi-quantified (C). New bone formation was analyzed by fluorochrome labeling (E) with calcein (green) and alizarin complexone (red) as well as the mineral apposition rate (MAR) between week 4 and 6 (D). (F and G) The bone mass quality of tibia was analyzed by micro-CT scanning at week 8. (H) Tibia length at week 8. Scale bar: 20 μm in (B) and (E); 300 μm in (F). n = 7-8 per group. *p < 0.05, **p < 0.01 and ***p < 0.001 *vs*. DM+veh group. One-way or two-way ANOVA followed by Tukey posttest analysis.

NADPH oxidases (NOX), a ROS-generating enzyme family, are major sources of ROS in the vascular system 36, 37. ECs express four NOX isoforms in humans, namely NOX1, NOX2, NOX4, and NOX5 36. Studies on hypertension, diabetes, and atherosclerosis have suggested that NOX 1 and NOX2 are the main mediators of endothelial oxidative stress and dysfunctions, whereas NOX4 is vasoprotective in increasing nitric oxide bioavailability and suppressing cell death pathways 36, 38, 39. To investigate the role of NOX in the diabetes-induced endothelial oxidative stress in bone blood vessels, we first examined the expressions of these three NOX isoforms in mice tibia ECs. qPCR results showed that, for the mRNA expression in the bone ECs of control animals, NOX2 ranked first, followed by NOX4 and NOX1 (Figure S5C). Compared with control mice, diabetic mice showed significant up-regulation of NOX1 and NOX2, but not NOX4 (Figure 8A), in tibia ECs. The antioxidative enzyme superoxide dismutase 1, but not catalase, was also significantly increased in bone ECs in diabetic groups (Figure S5D). Following insulin treatment, the diabetes-induced changes of NOXs were significantly reversed (Figure 8A). Next, a NOX1/4 inhibitor, GKT137831 (GKT), two different NOX2 inhibitors, NOX2ds-tat (NOX2ds) and GSK2795039 (GSK), or a pan-NOX inhibitor, VAS2870 (VAS) were intraperitoneally administrated in diabetic mice from week 4 through 8, and the therapeutic effects evaluated. The NOX1/4 inhibitor, GKT, did not influence the levels of glucose and insulin in the blood (Figure 8B-C), body weight gain (Figure 8D), ROS level in ECs (Figure 8I), the number and functions of ECs (Figure 8E-H and Figure 9A) and osteoblasts (Figure 9B-H), and bone resorption (Figure S3G-I) in diabetic tibia. VAS only partly increased the number and functions of ECs (Figure 8E-H) and osteogenic cells (Figure 9A-E) in the tibia, while NOX2ds and GSK both significantly attenuated the oxidative stress of bone ECs, promoted the recovery of type H ECs and coupling of angiogenesis and osteogenesis, with GSK showing the most significant effects. Next, the effects of a half dose of GSK in combination with a low dose of insulin in DM mice were examined from week 4 to 8 (Figure 4-5). The combination of relatively low doses of GSK and insulin from week 4 through 8 produced significantly better therapeutic effects than insulin alone (DM + ins lo + GSK lo 4-8w *vs*. DM + ins lo 4-8w).

## Discussion

Pathophysiological mechanisms underlying the poor bone formation in T1DM patients, leading to bone loss and frequent fragile fractures, remain unclear. Recent advances in our understanding of the organ-specific vascular system and endothelial cells (ECs) point to the injury of type H vessels in the bone as a possible important factor. In the present study, the main findings are as follows: First, early-onset T1DM leads to a holistic and irreversible abnormality of the vascular system, especially type H vessels in the bone, resulting in uncoupling of angiogenesis and osteogenesis and impaired bone growth; therefore, vascular injury might be a critical contributor to the increased bone fragility in patients with T1DM. Second, the T1DM-induced vascular lesions in the bone seem irreversible and related to glycemic levels (Figure S2), and early-started treatments, such as sufficient insulin therapy, are needed to control the disease. And third, endothelial oxidative stress, closely linked to the up-regulation of NOX2 in ECs, might be an important mechanism underlying the impairment of type H vessels in the diabetic bone, and NOX2 inhibitors, such as GSK2795039, show potential for clinical therapy of bone diseases in T1DM patients.

Vascular injury is a critical player in the dysfunctions of many organs afflicted by diabetes, known as vascular complications of diabetes 5, 40. However, the influence of diabetes on the vascular system in the bone and its role in diabetic osteopathy remained elusive. Previous studies on bone blood vessels in diabetes mainly concentrated on the relationship between vascular injury and abnormal hematopoiesis in bone marrow 7, 8, with very few focusing on the potential association between bone vascular system and bone mass lesions. There were controversies on the latter issue, originating from the contradictory data and the lack of explanatory rationale between vascular injury and diabetic osteopathy 7, 8.

In the present study, our results showed that juvenile T1DM led to significant reduction and dysfunction of type H vessels in the bone, leading to the uncoupling of angiogenesis and osteogenesis, resulting in compromised bone formation and fragile bone mass. These observations were in line with previous reports showing the bone marrow microangiopathy, reduced angiogenesis and bone turnover in T1DM mice 41, 42. Our results were also consistent with clinical observations in T1DM patients, showing that the subgroup with microvascular diseases had substantially severer bone loss than the subgroup without microvascular diseases despite similar glycemic control and insulin dosage 7, 8. These findings suggested an association between microvascular disease and bone mass abnormalities.

Recent research advances have furthered our comprehension of blood vessels and ECs that undergo organ-specialization. Tissue-specific endothelium establishes specialized vascular niches that deploy growth factors with angiocrine functions and play a critical role in the maintenance and regulation of development, homeostasis, and regeneration 9, 43. As organ-specialized vascular ECs, type H ECs are organized as interconnected micro-vessels in the metaphysis and near endosteum, controlling bone-forming cell lineage by releasing pro-osteogenic factors and mediating the coupling of angiogenesis and osteogenesis 11, 13. The proteolytic activity of type H endothelium has been shown to be essential for resorbing growth plate cartilage to lead directional bone growth and disrupting the orientation of type H vessels results in contorted bone shape 44. When the bone vascular system is affected by metabolic disturbances in diabetes, osteogenic cells might lose the support and regulation from type H vessels, resulting in impaired osteogenesis and increased bone fragility. To the best of our knowledge, our study is the first report about type H ECs under diabetic conditions, and provides a reasonable explanation for the causal link between vascular impairment and bone fragility. Together with previous reports, our results propose that diabetic osteopathy might be a chronic microvascular complication of T1DM 7.

Sustained high glycemic level and metabolic disturbance resulting from absolute deficiency of insulin are the core pathogenetic factors of T1DM complications 45. Our results suggested a positive correlation between the vascular impairment intensity in the diabetic bone and glycemic level (Figures 1-4 and Figure S2), with the worse the blood glucose control, the severer the bone vascular injury. This is consistent with clinical findings that the presence of microvascular disease serves as a proxy for general disease severity or poor glycemic control 7. Preclinical reports have also indicated that glycemic control level impacts bone metabolism 46, suggesting the loss of glycemic control as a critical reason for vascular system dysfunctions in the bone, especially type H vessels.

In the present study, insulin treatment as the standard therapy for T1DM, induced discrepant effects when applied at two different stages of the disease or delivered in two different doses. The amelioration of osteopathy by early-started sufficient insulin treatment in our study was in accord with the bone-anabolic role of insulin 1 and efficacy of insulin therapy for bone disease in T1DM in clinical 1, 47 and preclinical 48, 49 studies. Kalyanaraman *et al*. reported that hyperglycemia 49. This provides a possible explanation for the ineffectiveness of late-started insulin treatment in the present study. Exposure to high glucose might impact insulin signaling in bone cells including ECs, desensitizing cells to exogenous insulin in the late stage of T1DM. These observations suggest that bone vessel lesions and bone mass in T1DM are irreversible and could be controlled only if glycemic control is applied early after the disease onset. Therefore, early screening for bone diseases in diabetic patients is required. The substantial link between diabetic microangiopathy and skeletal fragility also suggests the necessity for including diabetic microvascular complications, such as retinopathy, in fracture risk prediction methods, like the Fracture Risk Assessment (FRAX) tool(6).

Collective evidence indicates oxidative stress as a crucial contributor to the injuries of many tissues in diabetes 50, 51. Compared with other organs, however, the role of oxidative stress in diabetic bone diseases is still elusive 32, 52. ROS serves as a key signal in the bone marrow stem cells niche in physiological conditions, regulating cell behaviors and mediating intercellular dialogue 33, 53. In diabetes, however, bone marrow microenvironment has been shown to be fiercely stricken by the diabetes-induced excessive ROS 33. It has been proposed that the oxidative stress in bone marrow vessels significantly contributes to the compromised blood infusion and the disorganized redox signals in the hematopoietic microenvironment, resulting in the inhibition of hematopoietic and regenerative functions 33, 41, 54.

According to the recently identified vascular subtypes in the bone, the ECs involved in most previous studies seemed to be type L ECs which exist mainly in the diaphysis forming the niche for hematopoietic stem cells 13, 43. In the present study, focusing on the bone mass metabolism, our results implicated oxidative stress in the dysfunction of another bony endothelial subtype, namely type H ECs, responsible for the coupling of angiogenesis and osteogenesis 11. Together with recent reports showing the importance of oxidative stress in diabetic osteoblast injury 49, our findings suggested anti-oxidative treatments as a promising therapy for osteopathy in T1DM.

It has been reported that ROS is an important contributor to the inhibition of insulin-secretion from β cells and inflammatory M1 macrophage infiltration in islets, inducing T1DM. Our results confirmed that treatment with the antioxidant TPO induced increased insulin and reduced blood glucose in diabetic animals. However, the correlation analysis between the glycemic level and ratio of type H ECs (Figure S2) showed that the decreased glycemic level in the DM+TPO group could not lead to significant attenuation of vascular lesions in the diabetic bone. On the other hand, *in vitro* results (Figure S6) suggested that antioxidants might exert their beneficial effects on bone vasculature by directly ameliorating the high glucose-induced oxidative stress and the consequent disruption of signals like Hif-1α in ECs.

Antioxidative treatment has been regarded as an important therapeutic strategy for many vascular diseases 55. However, despite the evidence from preclinical studies showing the efficacy of antioxidative drugs, such as vitamin E, limited benefits were observed in most clinical studies 39. Alternatively, it has been proposed that preventing ROS formation by targeting the sources of their generation could be a more effective strategy for attenuating oxidative stress than scavenging these highly reactive molecules after they are formed 39. NOX are the major sources of intracellular ROS in the cells of vascular walls 37, 56. Among the NOX family members, NOX1 and NOX2 have emerged as the two main isoforms implicated in endothelial oxidative stress, inflammation, and apoptosis 39, 56. However, the most critical NOX isoform responsible for endothelial injuries varies among different diseases, vascular levels, and tissues 56. In diabetic osteopathy, the role of NOX and the key isoform involved in pathogenesis has not yet been investigated.

In the present study, the results showed that T1DM led to the NOX1 and NOX2 increase in bone ECs and only NOX2 inhibitors induced significant therapeutic effects, with NOX2ds-tat and GSK2795039 showing high efficiency while VAS2870 was inefficient. These results indicated that NOX2 might be the key isoform mediating the T1DM-induced oxidative stress of bony vascular endothelium, especially type H ECs. Similarly, NOX2 inhibitor GSK2795039, due to its superior efficacy, might possess a higher potential for clinical therapy (Figure 7) and exert additional effects in combination with insulin (Figure 4-5). Our results are discordant with Gray *et al.* 57 reporting that NOX1 plays a critical role in T1DM-induced oxidative stress of aortic endothelial cells and accelerated aortic atherosclerosis, but consistent with some other reports on the critical role of NOX2 in the oxidative stress of vascular ECs 58-60. These conflicting data suggest that the key isoforms of NOX involved in the endothelial oxidative stress in T1DM vary among different tissues and between macrovascular and microvascular lesions.

As a systemic metabolic disease, diabetes could eventually impact all cell types, indicating that vascular injury is not the only mechanism for diabetic osteopathy. Recent findings have indicated that osteoblasts and preosteoclasts direct angiogenesis of type H vessels through paracrine factors, establishing a bidirectional regulation framework for the coupling of angiogenesis and osteogenesis. It is reasonable to speculate that aberrant angiogenesis and osteogenesis in T1DM could interact as both cause and effect of each other, together resulting in compromised bone quality. Further studies with cell type-specific intervention are needed to clarify whether vascular impairment is a crucial reason for diabetic osteopathy. Although there is ample evidence regarding the impaired bone formation in T1DM, the bone resorption changes in diabetes remain less investigated, and conflicting results have been reported 2. Consistent with most clinical studies 1, our data showed a reduction of osteoclast activity and bone resorption in the STZ-induced T1DM mouse model. This suggests that antiresorptive agents (e.g. bisphosphonates) might not be an appropriate treatment for bone impairment by T1DM and needs further investigation. Finally, the findings from animal models, need to be verified in human samples, and clinical analysis of human bone specimens should be conducted in the future.

## Conclusions

Herein, we demonstrated that the T1DM-induced irreversible injury of blood vessels in the bone, especially type H vessels, might be a critical contributor to the impaired bone formation and frequent fragile fractures in patients with early-onset T1DM. Our data furnished new evidence for the possibility that osteopathy could be a micro-vascular complication of diabetes. The extent of vascular lesions in diabetic bone was related to the glycemic level, and thus early disease screening and early-started treatments, such as sufficient insulin therapy, are needed to control the pathological progression. NOX2-mediated endothelial oxidative stress was an important player in the dysfunctions of type H vessels in diabetic bones. Therefore, inhibiting NOX2 might be a promising therapeutic strategy for bony vasculature impairments and bone mass in T1DM patients.

## Supplementary Material

Supplementary figures and tables.

## Abbreviations

T1DM: type 1 diabetes mellitus
EC: endothelial cell
NOX: NADPH oxidase
ROS: reactive oxygen species

## References

[1] Napoli N, Chandran M, Pierroz DD, Abrahamsen B, Schwartz AV, Ferrari SL (2017). Mechanisms of diabetes mellitus-induced bone fragility. Nat Rev Endocrinol.

[2] Sellmeyer DE, Civitelli R, Hofbauer LC, Khosla S, Lecka-Czernik B, Schwartz AV (2016). Skeletal Metabolism, Fracture Risk, and Fracture Outcomes in Type 1 and Type 2 Diabetes. Diabetes.

[3] Chen SC, Shepherd S, McMillan M, McNeilly J, Foster J, Wong SC (2019). Skeletal Fragility and Its Clinical Determinants in Children With Type 1 Diabetes. J Clin Endocrinol Metab.

[4] Weber DR, Schwartz G (2016). Epidemiology of Skeletal Health in Type 1 Diabetes. Curr Osteoporos Rep.

[5] Beckman JA, Creager MA (2016). Vascular Complications of Diabetes. Circ Res.

[6] Feng W, Liu S, Zhang C, Xia Q, Yu T, Zhu D (2019). Comparison of cerebral and cutaneous microvascular dysfunction with the development of type 1 diabetes. Theranostics.

[7] Shanbhogue VV, Hansen S, Frost M, Brixen K, Hermann AP (2017). Bone disease in diabetes: another manifestation of microvascular disease?. Lancet Diabetes Endocrinol.

[8] Fajardo RJ (2017). Is Diabetic Skeletal Fragility Associated with Microvascular Complications in Bone?. Curr Osteoporos Rep.

[9] Rafii S, Butler JM, Ding BS (2016). Angiocrine functions of organ-specific endothelial cells. Nature.

[10] Peng Y, Wu S, Li Y, Crane JL (2020). Type H blood vessels in bone modeling and remodeling. Theranostics.

[11] Kusumbe AP, Ramasamy SK, Adams RH (2014). Coupling of angiogenesis and osteogenesis by a specific vessel subtype in bone. Nature.

[12] Wang L, Zhou F, Zhang P, Wang H, Qu Z, Jia P (2017). Human type H vessels are a sensitive biomarker of bone mass. Cell Death Dis.

[13] Ramasamy SK, Kusumbe AP, Itkin T, Gur-Cohen S, Lapidot T, Adams RH (2016). Regulation of Hematopoiesis and Osteogenesis by Blood Vessel-Derived Signals. Annu Rev Cell Dev Biol.

[14] Xu R, Yallowitz A, Qin A, Wu Z, Shin DY, Kim JM (2018). Targeting skeletal endothelium to ameliorate bone loss. Nat Med.

[15] Huang J, Yin H, Rao SS, Xie PL, Cao X, Rao T (2018). Harmine enhances type H vessel formation and prevents bone loss in ovariectomized mice. Theranostics.

[16] Iyer S, Han L, Ambrogini E, Yavropoulou M, Fowlkes J, Manolagas SC (2017). Deletion of FoxO1, 3, and 4 in Osteoblast Progenitors Attenuates the Loss of Cancellous Bone Mass in a Mouse Model of Type 1 Diabetes. J Bone Miner Res.

[17] Wilcox CS (2010). Effects of tempol and redox-cycling nitroxides in models of oxidative stress. Pharmacol Ther.

[18] Aoyama T, Paik YH, Watanabe S, Laleu B, Gaggini F, Fioraso-Cartier L (2012). Nicotinamide adenine dinucleotide phosphate oxidase in experimental liver fibrosis: GKT137831 as a novel potential therapeutic agent. Hepatology.

[19] Altenhofer S, Kleikers PW, Radermacher KA, Scheurer P, Rob Hermans JJ, Schiffers P (2012). The NOX toolbox: validating the role of NADPH oxidases in physiology and disease. Cell Mol Life Sci.

[20] Jacobson GM, Dourron HM, Liu J, Carretero OA, Reddy DJ, Andrzejewski T (2003). Novel NAD(P)H oxidase inhibitor suppresses angioplasty-induced superoxide and neointimal hyperplasia of rat carotid artery. Circ Res.

[21] Ghosh J, Das J, Manna P, Sil PC (2010). Hepatotoxicity of di-(2-ethylhexyl)phthalate is attributed to calcium aggravation, ROS-mediated mitochondrial depolarization, and ERK/NF-kappaB pathway activation. Free Radic Biol Med.

[22] Khan MP, Singh AK, Joharapurkar AA, Yadav M, Shree S, Kumar H (2015). Pathophysiological Mechanism of Bone Loss in Type 2 Diabetes Involves Inverse Regulation of Osteoblast Function by PGC-1alpha and Skeletal Muscle Atrogenes: AdipoR1 as a Potential Target for Reversing Diabetes-Induced Osteopenia. Diabetes.

[23] Bouxsein ML, Boyd SK, Christiansen BA, Guldberg RE, Jepsen KJ, Muller R (2010). Guidelines for assessment of bone microstructure in rodents using micro-computed tomography. J Bone Miner Res.

[24] Goldschlager T, Abdelkader A, Kerr J, Boundy I, Jenkin G (2010). Undecalcified bone preparation for histology, histomorphometry and fluorochrome analysis. J Vis Exp.

[25] Yang M, Li CJ, Sun X, Guo Q, Xiao Y, Su T (2017). MiR-497 approximately 195 cluster regulates angiogenesis during coupling with osteogenesis by maintaining endothelial Notch and HIF-1alpha activity. Nat Commun.

[26] Patterson C, Guariguata L, Dahlquist G, Soltesz G, Ogle G, Silink M (2014). Diabetes in the young - a global view and worldwide estimates of numbers of children with type 1 diabetes. Diabetes Res Clin Pract.

[27] Wierzbicka E, Swiercz A, Pludowski P, Jaworski M, Szalecki M (2018). Skeletal Status, Body Composition, and Glycaemic Control in Adolescents with Type 1 Diabetes Mellitus. J Diabetes Res.

[28] Mayer-Davis EJ, Lawrence JM, Dabelea D, Divers J, Isom S, Dolan L (2017). Incidence Trends of Type 1 and Type 2 Diabetes among Youths, 2002-2012. N Engl J Med.

[29] Sivaraj KK, Adams RH (2016). Blood vessel formation and function in bone. Development.

[30] Filipowska J, Tomaszewski KA, Niedzwiedzki L, Walocha JA, Niedzwiedzki T (2017). The role of vasculature in bone development, regeneration and proper systemic functioning. Angiogenesis.

[31] Faria A, Persaud SJ (2017). Cardiac oxidative stress in diabetes: Mechanisms and therapeutic potential. Pharmacol Ther.

[32] Bacevic M, Brkovic B, Albert A, Rompen E, Radermecker RP, Lambert F (2017). Does Oxidative Stress Play a Role in Altered Characteristics of Diabetic Bone? A Systematic Review. Calcif Tissue Int.

[33] Mangialardi G, Spinetti G, Reni C, Madeddu P (2014). Reactive oxygen species adversely impacts bone marrow microenvironment in diabetes. Antioxid Redox Signal.

[34] Semenza GL (2014). Hypoxia-inducible factor 1 and cardiovascular disease. Annu Rev Physiol.

[35] Xiao H, Gu Z, Wang G, Zhao T (2013). The possible mechanisms underlying the impairment of HIF-1alpha pathway signaling in hyperglycemia and the beneficial effects of certain therapies. Int J Med Sci.

[36] Drummond GR, Sobey CG (2014). Endothelial NADPH oxidases: which NOX to target in vascular disease?. Trends Endocrinol Metab.

[37] Garcia-Redondo AB, Aguado A, Briones AM, Salaices M (2016). NADPH oxidases and vascular remodeling in cardiovascular diseases. Pharmacol Res.

[38] Gorin Y, Block K (2013). Nox as a target for diabetic complications. Clin Sci (Lond).

[39] Drummond GR, Selemidis S, Griendling KK, Sobey CG (2011). Combating oxidative stress in vascular disease: NADPH oxidases as therapeutic targets. Nat Rev Drug Discov.

[40] Sharma H, Lencioni M, Narendran P (2019). Cardiovascular disease in type 1 diabetes. Cardiovasc Endocrinol Metab.

[41] Oikawa A, Siragusa M, Quaini F, Mangialardi G, Katare RG, Caporali A (2010). Diabetes mellitus induces bone marrow microangiopathy. Arterioscler Thromb Vasc Biol.

[42] Peng J, Hui K, Hao C, Peng Z, Gao QX, Jin Q (2016). Low bone turnover and reduced angiogenesis in streptozotocin-induced osteoporotic mice. Connect Tissue Res.

[43] Potente M, Makinen T (2017). Vascular heterogeneity and specialization in development and disease. Nat Rev Mol Cell Biol.

[44] Romeo SG, Alawi KM, Rodrigues J, Singh A, Kusumbe AP, Ramasamy SK (2019). Endothelial proteolytic activity and interaction with non-resorbing osteoclasts mediate bone elongation. Nat Cell Biol.

[45] Zimmet P, Alberti KG, Magliano DJ, Bennett PH (2016). Diabetes mellitus statistics on prevalence and mortality: facts and fallacies. Nat Rev Endocrinol.

[46] Barmpa E, Vlychou M, Tigas S, Koukoulis GN, Bargiota A (2018). Impact of Glycaemic Control on Bone Metabolism in Adult Patients with Type 1 Diabetes Mellitus. Diabetes.

[47] Gilbert MP, Pratley RE (2015). The impact of diabetes and diabetes medications on bone health. Endocr Rev.

[48] Nyman JS, Kalaitzoglou E, Clay Bunn R, Uppuganti S, Thrailkill KM, Fowlkes JL (2017). Preserving and restoring bone with continuous insulin infusion therapy in a mouse model of type 1 diabetes. Bone Rep.

[49] Kalyanaraman H, Schwaerzer G, Ramdani G, Castillo F, Scott BT, Dillmann W (2018). Protein Kinase G Activation Reverses Oxidative Stress and Restores Osteoblast Function and Bone Formation in Male Mice With Type 1 Diabetes. Diabetes.

[50] Newsholme P, Cruzat VF, Keane KN, Carlessi R, de Bittencourt PI Jr (2016). Molecular mechanisms of ROS production and oxidative stress in diabetes. Biochem J.

[51] Volpe CMO, Villar-Delfino PH, Dos Anjos PMF, Nogueira-Machado JA (2018). Cellular death, reactive oxygen species (ROS) and diabetic complications. Cell Death Dis.

[52] Hamada Y, Fujii H, Fukagawa M (2009). Role of oxidative stress in diabetic bone disorder. Bone.

[53] Urao N, Ushio-Fukai M (2013). Redox regulation of stem/progenitor cells and bone marrow niche. Free Radic Biol Med.

[54] Fadini GP, Ciciliot S, Albiero M (2017). Concise Review: Perspectives and Clinical Implications of Bone Marrow and Circulating Stem Cell Defects in Diabetes. Stem Cells.

[55] Poprac P, Jomova K, Simunkova M, Kollar V, Rhodes CJ, Valko M (2017). Targeting Free Radicals in Oxidative Stress-Related Human Diseases. Trends Pharmacol Sci.

[56] Konior A, Schramm A, Czesnikiewicz-Guzik M, Guzik TJ (2014). NADPH oxidases in vascular pathology. Antioxid Redox Signal.

[57] Gray SP, Di Marco E, Okabe J, Szyndralewiez C, Heitz F, Montezano AC (2013). NADPH oxidase 1 plays a key role in diabetes mellitus-accelerated atherosclerosis. Circulation.

[58] Angioni R, Liboni C, Herkenne S, Sanchez-Rodriguez R, Borile G, Marcuzzi E (2020). CD73(+) extracellular vesicles inhibit angiogenesis through adenosine A2B receptor signalling. J Extracell Vesicles.

[59] Bendall JK, Rinze R, Adlam D, Tatham AL, de Bono J, Wilson N (2007). Endothelial Nox2 overexpression potentiates vascular oxidative stress and hemodynamic response to angiotensin II: studies in endothelial-targeted Nox2 transgenic mice. Circ Res.

[60] Fan LM, Geng L, Cahill-Smith S, Liu F, Douglas G, McKenzie CA (2019). Nox2 contributes to age-related oxidative damage to neurons and the cerebral vasculature. J Clin Invest.

