# Supplementary material for: Impairment of type H vessels by NOX2-mediated endothelial oxidative stress: critical mechanisms and therapeutic targets for bone fragility in streptozotocin-induced type 1 diabetic mice
Source: Theranostics. 2021 Jan 30;11(8):3796–812. doi: 10.7150/thno.50907 (PMC7914348; doi:10.7150/thno.50907)
Supplement: Supplementary file 1 — Supplementary figures and tables. [file thnov11p3796s1.pdf]

Supplementary Materials

Impairment of type H vessels by NOX2-mediated endothelial oxidative stress: critical mechanisms and therapeutic targets for bone fragility in streptozotocin-induced type 1 diabetic mice

Xiao-Fan Hu, Geng Xiang, Tian-Ji Wang, Yu-Bo Ma, Yang Zhang, Ya-Bo Yan, Xiong Zhao, Zi-Xiang Wu, Ya-Fei Feng, Wei Lei

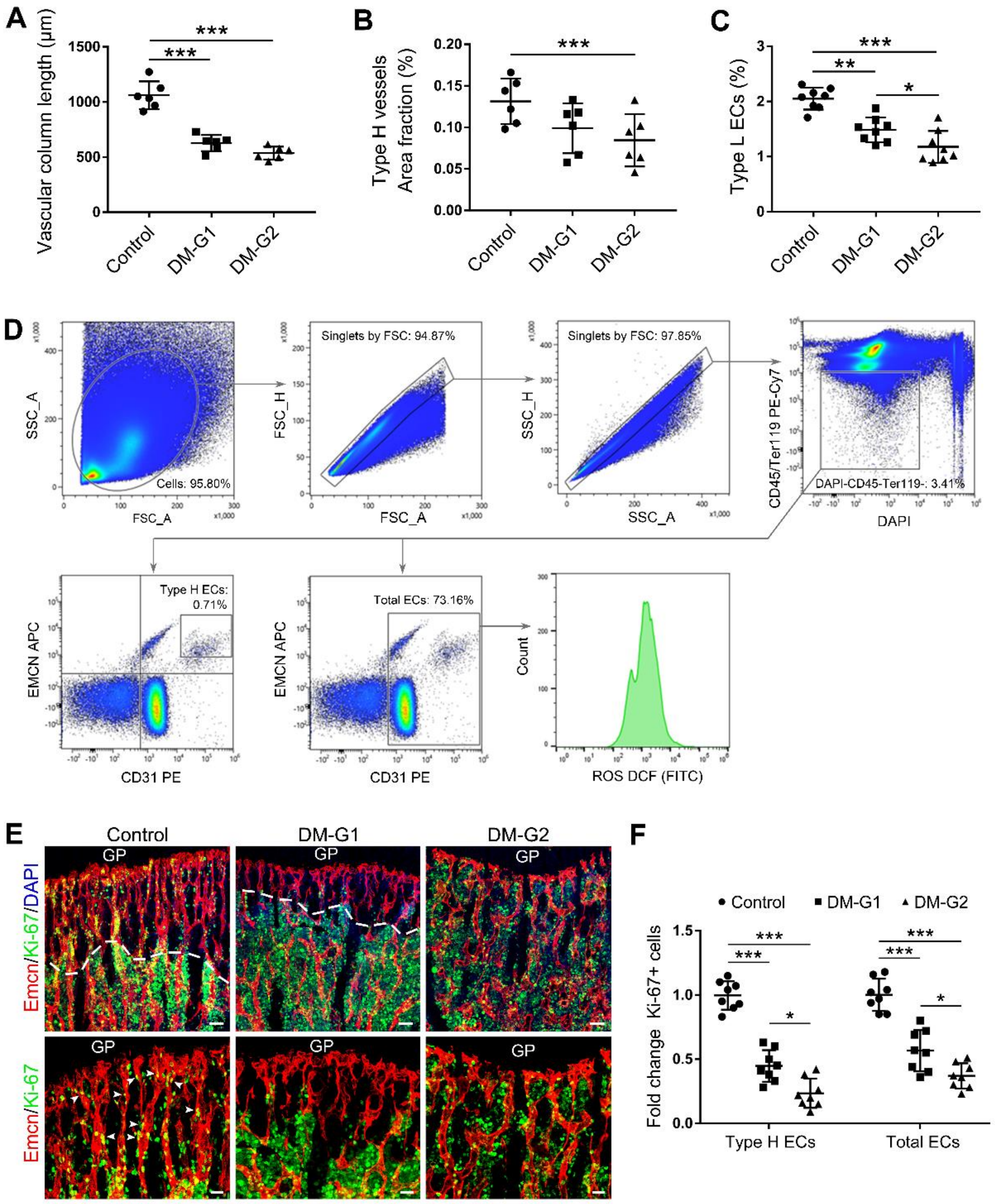

Figure S1. Effects of T1DM on vascular endothelial cells (ECs) in bone. Histomorphometry analysis of images represented by Fig. 1D as the length of vascular column under growth plate (A) and Fig. 1I as the area fraction of type H vessels near endosteum (B). (C) Flow cytometric quantitation of type L ECs in 4-week-old tibia. (D) Schematic representation of the strategy used in flow cytometry for analysis of CD31^{high}EMCN^{high} (type H) and total ECs in bone. (E) Confocal images showing the Emcn^{+} (red) endothelium and the Ki-67^{+} (green, proliferation marker) cell nuclei in the metaphysis of 4-week-old tibia. With the rise of glycemic level, the Ki-67^{+} ECs (arrowheads) decreased and the transition zone in metaphysis (above the dashed lines) between bone marrow and growth plate (GP) became narrower. Scale bar: 50 μm on

the above and 20 μm on the below. (F) Fold change in the Ki-67^+ type H ECs and total ECs numbers in 4-week tibia, determined by flow cytometry. Data are present mean ± SD. n = 6-8 per group. *$p < 0.05$ and ***$p < 0.001$. One-way ANOVA followed by Tukey posttest analysis.

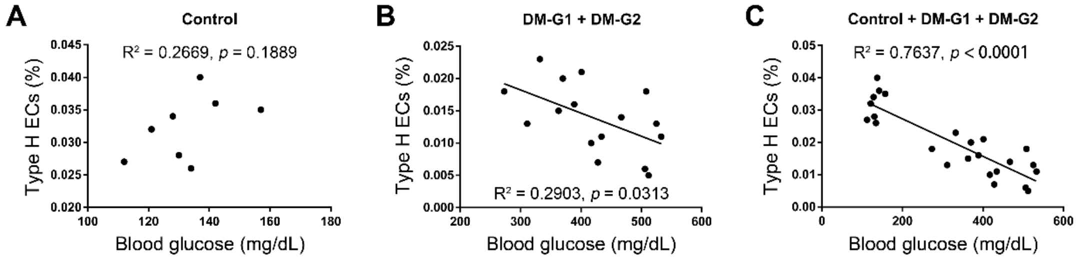

Figure S2. Correlation between glycemic level and the ratio of type H ECs in tibia at week 4. Correlation analysis and linear regression of the individual data show that the ratio of type H ECs is negatively related with glycemic level in DM groups, but not in control group.

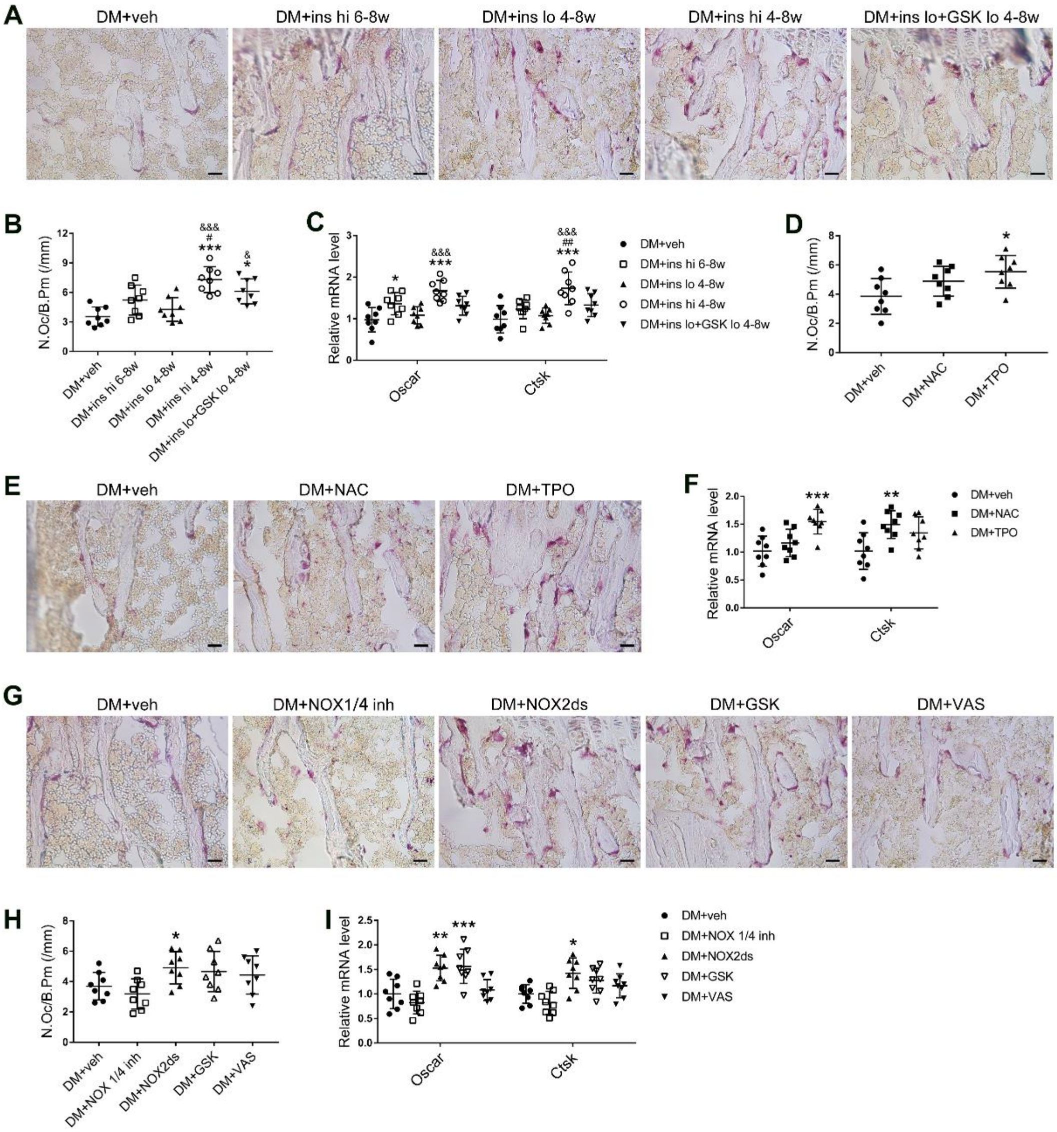

Figure S3. Effects of drugs on osteoclasts in diabetic tibia at week 8. (A, E and G) Images of TRAP-stained tibia sections. (B, D and H) Histomorphometric analysis of the number of osteoclasts per bone perimeter (N.Oc/B.Pm.). (C, F and I) qPCR analysis of the markers for osteoclast differentiation (Oscar, osteoclast associated receptor) and function (Ctsk, cathepsin K). n = 7-8 per group. *$p < 0.05$, **$p < 0.01$ and ***$p < 0.001$ *vs*. DM+veh group; $^{\#}p < 0.05$ and $^{\#\#}p < 0.01$ *vs*. DM+ins hi 6-8w group; $^{\&\&\&}p < 0.001$ *vs*. DM+ins lo 3-8w group. One-way or two-way ANOVA followed by Tukey posttest analysis.

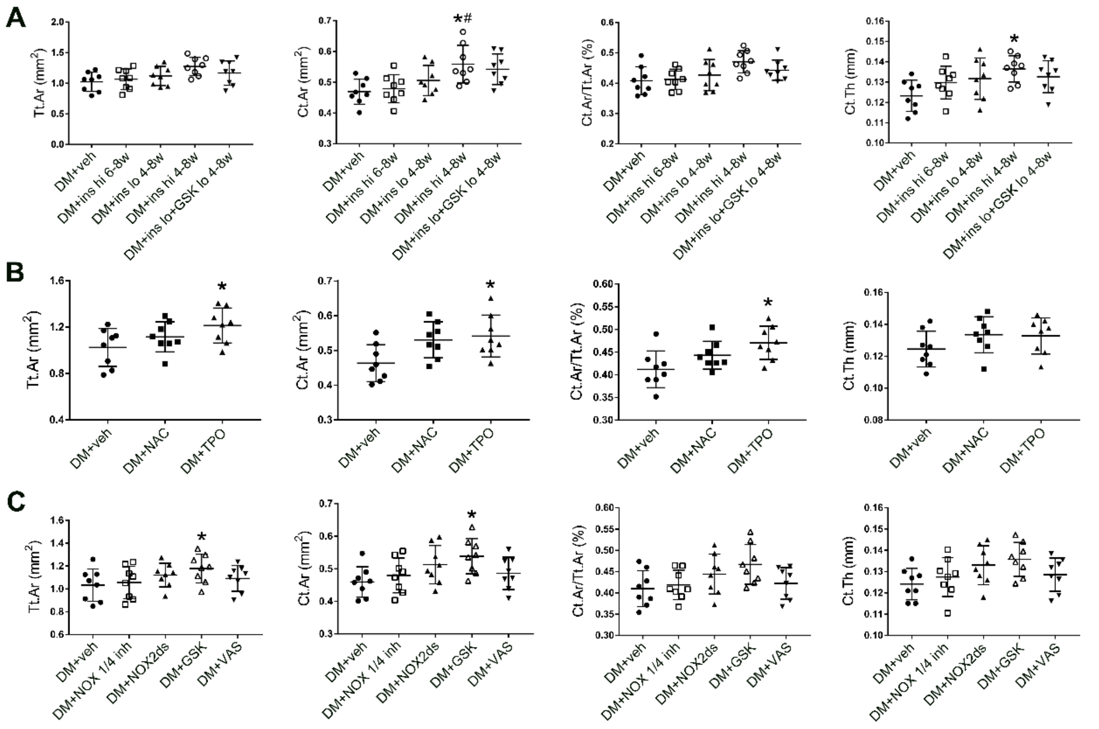

Figure S4. Geometry parameters of the cortical bone of tibia by micro-CT analysis at week 8. Tt.Ar, Total cross-sectional area inside the periosteal envelope; Ct.Ar, cortical bone area; Ct.Ar/Tt.Ar, cortical bone area fraction; Ct.Th, average cortical thickness. n = 7-8 per group. *$p < 0.05$ *vs*. DM+veh group; $^{\#}p < 0.05$ *vs*. DM+ins hi 6-8w group. One-way ANOVA followed by Tukey posttest analysis.

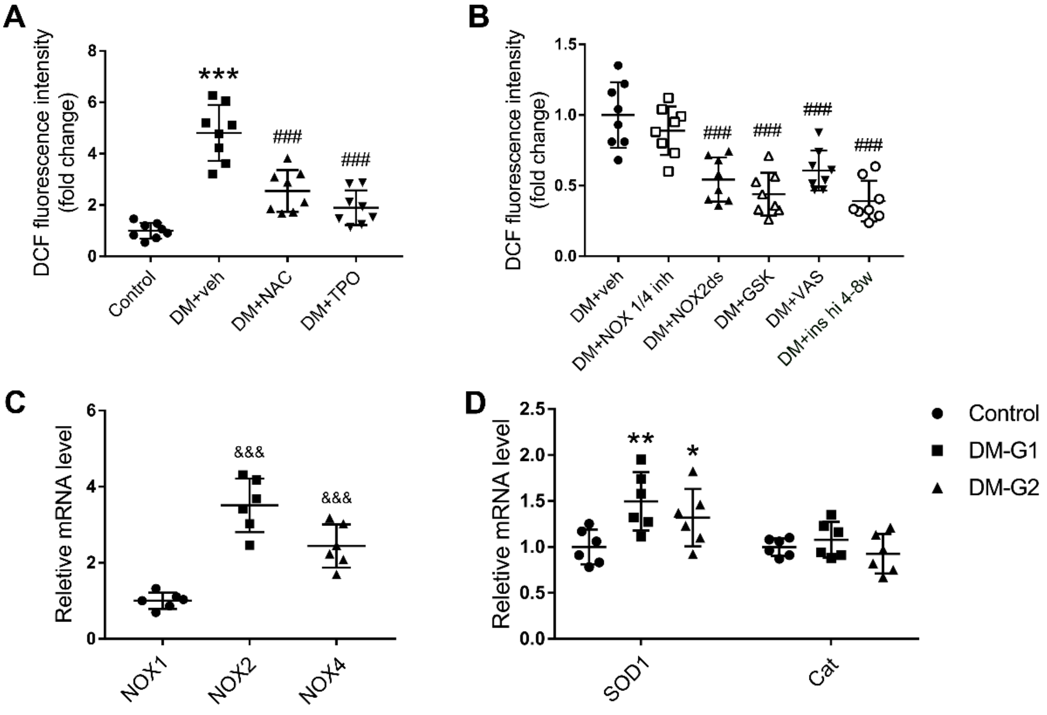

Figure S5. Redox metabolism in the ECs in tibia. (A and B) ROS levels in the type H ECs in 8-week-old tibia detected by flow cytometry analysis of intracellular DCF fluorescence. (C) qPCR analysis the mRNA expressions of three NOX isoforms in the bone ECs of control animals. (D) mRNA levels of Superoxide dismutase 1 (SOD1) and Catalase (Cat) in the bone ECs in different groups. $^{*}p < 0.05$, $^{**}p < 0.01$ and $^{***}p < 0.001$ *vs*. Control group; $^{\#\#\#}p < 0.001$ *vs*. DM+veh group; $^{\&\&\&}p < 0.001$ *vs*. NOX1 group. One-way ANOVA followed by Tukey posttest analysis.

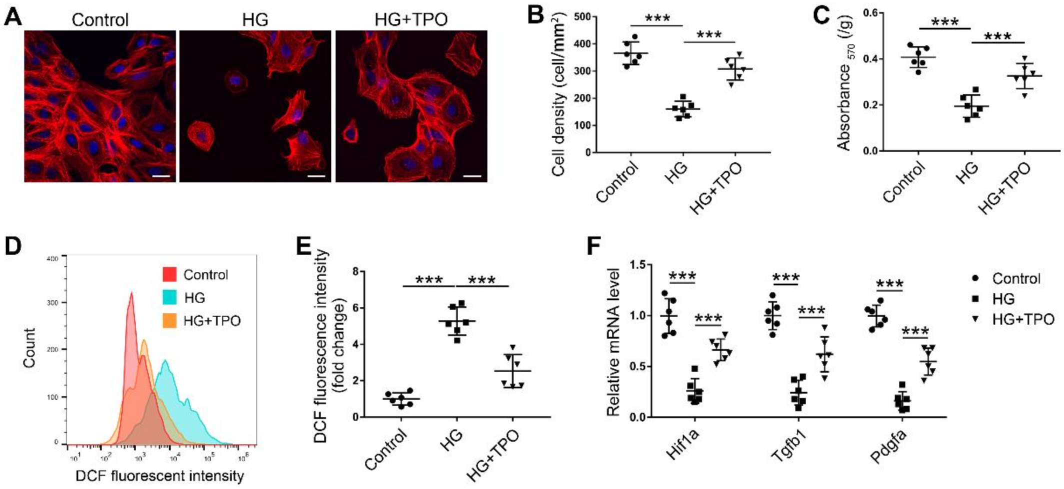

Figure S6. The effects of high glucose (HG) and antioxidant treatment on bone-specific ECs in vitro. Cells were tested after 4 days of incubation. (A) Representative fluorescent images show the cytoskeleton (red) and cell nuclei (blue) of ECs. (B) Quantitation of cell density in images as shown in (A). (C) Cell viability determined by colorimetric methylthiazol tetrazolium (MTT) assay. Representative histogram (D) and quantitation (E) in flow cytometry analysis show the DCH fluorescent intensity (ROS level) in ECs. (F) qPCR analysis of the expression of three molecules in ECs. ***$p <$ 0.001. One-way ANOVA followed by Tukey posttest analysis.

Supplemental Table 1. HbA1c in different groups at week 8.

Group	HbA1c (%, mean ± SD)	HbA1c (mmol/mol, mean)
Control	4.06 ± 0.55	21
DM-G1	8.36 ± 1.55*	68
DM-G2	10.80 ± 1.68*#	95
DM + veh	12.07 ± 1.04	108
DM + ins hi 6-8w	9.01 ± 1.06†	75
DM + ins lo 4-8w	10.2 ± 1.27†	87
DM + ins hi 4-8w	6.06 ± 1.61†‡§	43
DM + ins lo + GSK lo 4-8w	9.83 ± 1.24†	84
DM + veh	11.39 ± 0.93	100
DM + NAC	10.84 ± 0.74	95
DM + TPO	10.48 ± 1.02	91
DM + veh	11.63 ± 1.05	103
DM + NOX 1/4 inh	11.14 ± 0.97	98
DM + NOX2ds	10.55 ± 1.01	91
DM + GSK	10.04 ± 1.12†	86
DM + VAS	11.27 ± 0.99	99

The HbA1c mean values in % were converted into values in mmol/mol by the NGSP's HbA1c converter at http://www.ngsp.org/convert1.asp. n = 14-16 per group. *$p < 0.05$ *vs*. Control group; #$p < 0.05$ *vs*. DM-G1 group; †$p < 0.05$ *vs*. DM+veh group; ‡$p < 0.05$ *vs*. DM+ins hi 6-8w group; §$p < 0.05$ *vs*. DM+ins lo 4-8w group. One-way ANOVA followed by Tukey posttest analysis.

Supplemental Table 2. Primer Sequences Used for qPCR

Abbrev	Gene name	Forward primer sequence (5'-3')	Reverse primer sequence (5'-3')
Emcn	endomucin	AATACCAGGCATCGTGTCAGT	CCACTTCATGTTTTGGTGTTGTC
Pecam1	platelet/endothelial cell adhesion molecule 1	ACGCTGGTGCTCTATGCAAG	TCAGTTGCTGCCCATTCATCA
Nog	noggin	GCCAGCACTATCTACACATCC	GCGTCTCGTTCAGATCCTTCTC
Bmp2	bone morphogenetic protein 2	GGGACCCGCTGTCTTCTAGT	TCAACTCAAATTCGCTGAGGAC
Pdgfa	platelet derived growth factor, alpha	TGGCTCGAAGTCAGATCCACA	TTCTCGGGCACATGGTTAATG
Tgfb1	transforming growth factor, beta 1	CCACCTGCAAGACCATCGAC	CTGGCGAGCCTTAGTTTGGAC
Fgf1	fibroblast growth factor 1	GGGGAGATCACAACCTTCGC	GTCCCTTGTCCCATCCACG
Wnt3a	wingless-type MMTV integration site family, member 3A	CTCCTCTCGGATACCTCTTAGTG	CCAAGGACCACCAGATCGG
Bglap	bone gamma carboxyglutamate protein (osteocalcin)	TTTCTGCTCACTCTGCTGACC	TGCTTGGACATGAAGGCTTTG
Ibsp	integrin binding sialoprotein	GGACTGCCGAAAGGAAGGTT	CCGGTACTTAAAGACCCCGTTT
Ctsk	cathepsin K	GAAGAAGACTCACCAGAAGCAG	TCCAGGTTATGGGCAGAGATT
Oscar	osteoclast associated receptor	CCTAGCCTCATACCCCCAG	CGTTGATCCCAGGAGTCACAA
Hif1a	hypoxia inducible factor 1, alpha subunit	ACCTTCATCGGAAACTCCAAAG	ACTGTTAGGCTCAGGTGAACT
Nox1	NADPH oxidase 1	GGTTGGGGCTGAACATTTTTC	TCGACACACAGGAATCAGGAT
Nox2	cytochrome b-245, beta polypeptide (Cybb)	CCTCTACCAAAACCATTCGGAG	CTGTCCACGTACAATTCGTTCA
Nox4	NADPH oxidase 4	GAAGGGGTTAAACACCTCTGC	ATGCTCTGCTTAAACACAATCCT
SOD1	superoxide dismutase 1	AACCAGTTGTGTTGTCAGGAC	CCACCATGTTTCTTAGAGTGAGG
Cat	catalase	AGCGACCAGATGAAGCAGTG	TCCGCTCTCTGTCAAAGTGTG
Actb	actin beta	GTGACGTTGACATCCGTAAAGA	GCCGGACTCATCGTACTCC
